# Pulmonary Response to Surface-Coated Nanotitanium Dioxide Particles Includes Induction of Acute Phase Response Genes, Inflammatory Cascades, and Changes in MicroRNAs: A Toxicogenomic Study

**DOI:** 10.1002/em.20639

**Published:** 2011-01-21

**Authors:** Sabina Halappanavar, Petra Jackson, Andrew Williams, Keld A Jensen, Karin S Hougaard, Ulla Vogel, Carole L Yauk, Håkan Wallin

**Affiliations:** 1 Environmental Health Science and Research BureauHealth Canada, Ottawa, Ontario, Canada; 2 National Research Centre for the Working EnvironmentCopenhagen, Denmark; 3 Institute for Science, Systems and Models, Roskilde UniversityRoskilde, Denmark; 4 National Food Institute, Technical University of DenmarkSøborg, Denmark; 5 Institute of Public Health, University of CopenhagenCopenhagen, Denmark

**Keywords:** nanotitanium dioxide, gene expression, microRNA, inflammation

## Abstract

Titanium dioxide nanoparticles (nanoTiO_2_) are used in various applications including in paints. NanoTiO_2_ inhalation may induce pulmonary toxicity and systemic effects. However, the underlying molecular mechanisms are poorly understood. In this study, the effects of inhaled surface-coated nanoTiO_2_ on pulmonary global messenger RNA (mRNA) and microRNA (miRNA) expression in mouse were characterized to provide insight into the molecular response. Female C57BL/6BomTac mice were exposed for 1 hr daily to 42.4 ± 2.9 (SEM) mg surface-coated nanoTiO_2_/m^3^ for 11 consecutive days by inhalation and were sacrificed 5 days following the last exposure. Physicochemical properties of the particles were determined. Pulmonary response to nanoTiO_2_ was characterized using DNA microarrays and pathway-specific PCR arrays and related to data on pulmonary inflammation from bronchial lavages. NanoTiO_2_ exposure resulted in increased levels of mRNA for acute phase markers *serum amyloid A-1 (Saa1)* and *serum amyloid A-3 (Saa3*), several C-X-C and C-C motif chemokines, and cytokine *tumor necrosis factor* genes. Protein analysis of Saa1 and 3 showed selective upregulation of Saa3 in lung tissues. Sixteen miRNAs were induced by more than 1.2-fold (adjusted *P*-value < 0.05) following exposure. Real time polymerase chain reaction confirmed the upregulation of miR-1, miR-449a and revealed dramatic induction of miR-135b (60-fold). Thus, inhalation of surface-coated nanoTiO_2_ results in changes in the expression of genes associated with acute phase, inflammation and immune response 5 days post exposure with concomitant changes in several miRNAs. The role of these miRNAs in pulmonary response to inhaled particles is unknown and warrants further research. Environ. Mol. Mutagen., 2011. © 2011 Wiley-Liss, Inc.†

## BACKGROUND

Rapid developments in nanotechnology are resulting in increased use and potential release of a variety of engineered nanomaterials into the workplace and environment. Nanoparticles (NPs) are particles with a diameter less than 100 nm along at least one dimension and exhibit new or enhanced size-associated properties compared with larger particles of the same material. The large surface area, unique surface chemistry, and reactivity of NPs pose a unique challenge in assessing their effects on biological systems. Consequently, there is little knowledge of the molecular mechanisms leading to toxic effects following exposure to the diverse types of NPs, and there is no accepted framework for risk assessment of NP exposure [Oberdorster et al.,2005a,b; Borm et al.,^2006^].

Titanium dioxide nano particles (nanoTiO_2_) are poorly soluble and were originally considered to be physiologically inert, posing little risk to human health. However, the International Agency for Research on Cancer has recently classified pigment-grade titanium dioxide as a group 2B carcinogen [IARC, Feb.^2006^]. NanoTiO_2_ has also been listed as a high priority NP by the Organization for Economic Cooperation and Development steering group for test guidelines. NanoTiO_2_ are widely used in paints, paper, plastics, ceramics, and cosmetic sunscreens. Thus, there is potential for human exposure to nanoTiO_2_ occupationally or through release from consumer products. As a result, there is an urgent need for experimental investigations on the biological effects of various types of NPs to increase our understanding of the mechanisms of NP action and establish safe regulatory guidelines for human exposure.

Studies have demonstrated that nanoTiO_2_ causes cellular damage in exposed rodents. For example, chronic exposure to high doses of nanoTiO_2_ by inhalation causes bronchoalveolar hyperplasia, metaplasia, pulmonary fibrosis, and tumor formation in rats [Lee et al.,^1985^; Heinrich et al.,^1995^], possibly resulting from impaired particle clearance [Warheit et al.,^1997^; Cullen et al.,^2000^]; lower doses cause lung inflammation in mice [Dankovic et al.,^2007^]. A single intratracheal dose of nanoTiO_2_ can disrupt alveolar septa and induce emphysema-like changes in mouse lungs [Chen et al.,^2006^]. Particle accumulation in lungs and interstitial pneumonia associated with alveolar septal thickening results following intraperitoneal injection of nanoTiO_2_ in mice [Chen et al.,^2009^]. A multispecies, subchronic, inhalation study demonstrated similar inflammatory responses, lung burdens, and pulmonary overload in both mice and rats exposed at high doses [Bermudez et al.,^2004^]. NanoTiO_2_ is also cytotoxic and causes DNA damage and mutation in various animal and human cell lines [Rahman et al.,^2002^; Bermudez et al.,^2004^; Sayes et al.,^2006^; Warheit et al.,^2006^; Wang et al.,^2007^].

To increase its stability in paints, lotion, or creams, nanoTiO_2_ in general are surface coated with silica, alumina, or other polymers. Surface-coated nanoTiO_2_ in sunscreen lotions is expected to reduce its photoactivity. However, surface coatings and functionalization play a major role in nanoTiO_2_-induced toxicity. For instance, mice challenged with nanoTiO_2_ coated with silica exhibited significantly larger inflammatory responses than mice challenged with uncoated nanoTiO_2_ or silica alone [Rossi et al.,^2010^]. Similarly, vanadium pentoxide-coated anatase nanoTiO_2_ particles induced more cytotoxicity and genotoxicity than natural anatase [Bhattacharya et al.,^2008^]. Therefore, nanoTiO_2_ toxicity is enhanced by surface modifications of the particles. However, it is unclear what changes arise at the molecular level that lead to differences in the toxicity induced by uncoated nanoTiO_2_ relative to particles that are surface modified.

Genomic analyses provide a means to analyze the entire transcriptome of cells or tissues [Mei et al.,^2010^]. Early changes in critical pathways may be used to predict eventual health outcomes as well as derive the molecular mechanisms leading to toxicity [Hanahan and Weinberg,^2000^; Bhattacharjee et al.,^2001^; Golpon et al.,^2004^; Ning et al.,^2004^; Spira et al.,^2004^; Granville and Dennis,^2005^]. However, mRNA expression does not always correlate with related protein abundance and activity, as mRNA and proteins may be post-transcriptionally regulated. Small noncoding RNAs known as microRNAs (miRNAs) have recently been found to play a central role in the regulation of gene expression and protein translation. MiRNAs are typically 21–25 nucleotides in length and function in translational repression or mRNA degradation via the RNA interference pathway [Kim et al.,^2009^; Winter et al.,^2009^]. MiRNAs are also thought to play an important role in the maintenance of chromatin structure and are therefore critical mediators of gene expression and genome stability [Guil and Esteller,^2009^]. MiRNAs are involved in numerous biological processes including apoptosis [Lynam-Lennon et al.,^2009^], cell cycle progression [Carleton et al.,^2007^; Bueno et al.,^2008^], development (reviewed in [Sun and Tsao,^2008^]), and immune response (reviewed in [Tsitsiou and Lindsay,^2009^]). Aberrant miRNA expression has been found in several human diseases (reviewed in [Wang et al.,^2008^]), and many cancers express unique miRNA signatures [Croce,^2009^; Garzon et al.,^2009^]. As such, disruption of miRNA expression is now being widely investigated and various techniques have been developed for this purpose (reviewed in [Kong et al.,^2009^]).

We have recently investigated the developmental and neurological effects of surface-coated nanoTiO_2_ particles [Hougaard et al.,^2010^]. We also reported pulmonary inflammation in nonpregnant adult female mice exposed by inhalation to a low, biologically relevant dose of rutile nanoTiO_2_ particles that were surface modified and coated [Hougaard et al.,^2010^]. In this study, we apply DNA microarrays, pathway-specific real-time polymerase chain reaction (RT-PCR) arrays, focussed RT-PCR, and protein analysis to characterize the molecular changes associated with the observed pulmonary inflammation in the same nonpregnant adult mice exposed to surface-coated nanoTiO_2_ particles from the above study.

## MATERIALS AND METHODS

### Material, Animal Handling, and Tissue Collection

This study used UV-titan L181 with particle size of 20 nm (Kemira, Pori, Finland) enriched in rutile and modified with amounts of zirconium, silicon, aluminum, and coated with polyalcohol.

The study was conducted in parallel with a developmental toxicity study and therefore the particle dose and the time point were selected accordingly. Detailed information about animals, exposure, and exposure monitoring is described by Hougaard et al. [2010]. Briefly, 45 time-mated, nulliparous, adult female C57BL/6BomTac mice were treated as described by Hougaard et al. [2008] by whole-body inhalation to 42.4 ± 2.9 (SEM) mg nanoTiO_2_/m^3^ or to filtered air for 1 hr/day for 11 days. Seventeen (nine controls and eight exposed) of these animals did not conceive (lack of pups and interuterine implantation sites) and were used in this study as exposed adult females.

Animals were sacrificed by cardiac puncture 5 days after the last exposure. Left lung and a section of the liver were sliced randomly into portions and used for (a) total RNA extraction; (b) total RNA extraction enriched with small RNAs; and (c) total protein extracts. Samples were stored at −80°C until analysis. All procedures complied with EC Directive 86/609/EEC and Danish laws regulating experiments on animals (permit 2006/561-1123).

### Material Characterization

Details of sample preparation and particle analysis are described in the Supporting Information provided by Hougaard et al. [2010]. In brief, physical particle size, morphology, and general state of agglomeration/aggregation were determined by transmission electron microscope (TEM, Tecnai G20, FEI Company, Hillsboro, OR). Crystalline phases and crystallite sizes were determined by powder X-ray diffraction (XRD) with a Bruker D8 Advance diffractometer equipped with a Lynxeye CCD detector (Bruker AXS, Madison, WI 53711-5373). Results were obtained by Rietveld refinement of the X-ray diffractograms using Bruker 10 TOPAS V4.1 software. Specific surface area was determined on a Quantachrome Autosorp-1 (Quantachrome GmbH & Co. KG, Odelzhausen, Germany) using multipoint Brunauer, Emmett, and Teller (BET) nitrogen adsorption method after 1 hr degassing at 300°C. Analysis was completed according to DIN ISO 9277 as a commercial service by Quantachrome GmbH & Co. KG. Elemental composition was analyzed by X-ray Fluorescence analysis on a Philips PW-2400 spectrometer as a commercial service by the Department of Earth Sciences, University of Aarhus, Denmark.

### Exposure Monitoring

The fine particle exposure (<600 nm) was monitored using a GRIMM Sequential (Stepping) Mobility Particle Sizer (SMPS, Model No. 5.521; Serial No. 5LP 10209) connected to a GRIMM Condensation Particle Counter (Model 5.400). The SMPS data sampling and calculations were completed using the GRIMM software 5.477/02 v. 1.34 and operated in the fast scan mode. Data were corrected for both Classifier and CPC efficiency using the available software options. Particles were neutralized using a 3.7 MBq Am-241 source (Model No. 5.521). Coarse particle exposure (0.75 to > 15 μm) was measured using a GRIMM Dust Monitor (Model 1.105) at a resolution of 6 sec. The Dust Monitor particle sizes were subsequently recalculated to geometric means assuming an upper channel cut point at 20 μm.

### Titanium in Tissue

Approximately 25–75 mg of lung tissue was weighed and digested in concentrated nitric acid (PlasmaPure, SCP Science, Quebec, Canada) in a microwave oven (Multiwave, Anton Paar, Graz, Austria), and titanium content was determined by quadrupole-based inductively coupled plasma mass spectrometer (ICPMS 7500ce, Agilent Technologies, Tokyo, Japan) equipped with a collision/reaction cell (CRC). The limit of detection (LOD) for titanium in tissues, based on three times the standard deviation of repeated blank measurements, was estimated to be 0.2–5 mg/kg depending on sample intake and dilution. Additional details are described by Hougaard et al. [2010].

### Total RNA and MiRNA Extraction and Purification

Total RNA was isolated from the lung and the liver (*n* = 8/group) using TRIzol reagent (Invitrogen) and purified using RNeasy Mini Kit (Qiagen). The mirVana miRNA Isolation Kit (Ambion, Streetsville, ON, Canada) was used to prepare total RNA enriched with small RNA species from randomly selected left lung sections. RNA quality was confirmed by UV spectrophotometry and using an Agilent bioanalyzer (Agilent Technologies).

### Microarray Hybridization

Individual total RNA (250 ng) samples from eight mice per treatment group (control or exposed) and universal reference total RNA (Stratagene) were used to synthesize double-stranded cDNA and cyanine labeled cRNA according to the manufacturer's instructions (Agilent Linear Amplification Kits, Agilent Technologies). Experimental samples were labeled with Cyanine 5-CTP and reference RNA with Cyanine 3-CTP (Perkin-Elmer Life Sciences). Cyanine-labeled cRNA targets were in vitro transcribed using T7 RNA polymerase, purified by RNeasy Mini Kit (Qiagen) and were hybridized to Agilent mouse 4 × 44 oligonucleotide microarrays (Agilent Technologies) at 60°C overnight. Arrays were washed and scanned on an Agilent G2505B scanner. Data were acquired using Agilent Feature Extraction software version 9.5.3.1.

### MiRNA Expression Profiling

Freshly isolated individual total lung RNA samples from eight control and eight treated samples were labeled using the Agilent miRNA Complete Labeling and Hybridization Kit (Agilent Technologies). Briefly, 100 ng of total RNA was dephosphorylated by incubation with calf intestinal phosphatase at 37°C for 30 min, denatured using 100% DMSO at 100°C for 5 min, then labeled with pCp-Cy3 using T4 ligase by incubation at 16°C for 1 hr. The labeled RNA samples were hybridized to an individual array on 8 × 15K format Agilent mouse miRNA array slides. Hybridizations were performed in SureHyb chambers (Agilent) at 55°C for 20 hrs. Arrays were washed, scanned at a resolution of 5 μm using an Agilent G2505B scanner and data were acquired using Agilent Feature Extraction software version 9.5.3.1.

### Statistical Analysis of Microarray Data

A reference design [Kerr,^2003^; Kerr and Churchill,^2007^] was used to analyze gene expression microarray data. Data were normalized using LOWESS in R [R-Development-Core-Team,^2004^] and differential expression was determined using MAANOVA [Wu et al.,^2003^]. The Fs statistic [Cui et al.,^2005^] was used to test for treatment effects, and *P*-values were estimated by the permutation method using residual shuffling, followed by adjustment for multiple comparisons using the false discovery rate (FDR) approach [Hochberg,^1995^]. Fold change calculations were based on the least-square means. Significant genes were identified as having an adjusted *P*-value < 0.05 for any individual contrast.

### Agilent MiRNA Microarray Analysis

Nonbackground subtracted raw data were quantile normalized [Bolstad et al.,^2003^]. Present calls were determined as signals that were >3 trimmed SDs above the trimmed mean of the (−)3 × SLv1 probes on the array. Probes with technical replicates for a miRNA were averaged using the median signal intensity. Boxplots and cluster analyses were used to identify potential outliers (poor quality chips). This quality control check resulted in the elimination of six arrays from the analysis. Identification of differentially expressed miRNA was carried out at the probe level as well as the miRNA level. The MAANOVA model included the sample identity as a random effect and the gene specific variance estimate (F1 Test) was used to test for differences between the control and treated samples. In this analysis, parametric *P*-values were obtained and were then FDR corrected.

### Real-Time Polymerase Chain Reaction

#### Microarray

Primers were designed using Beacon design 2.0 (Premier BioSoft International). Approximately 2.5 μg of total RNA per sample was reverse transcribed and RT-PCR was performed in duplicate using an iCycler IQ real-time detection system (Bio-Rad). Threshold cycle values were averaged. Gene expression levels were normalized to the *GAPDH* and *Hprt* gene, which were stable on the DNA microarray. PCR efficiency was examined using the standard curve for each gene. Primer specificity was assured by the melting curve for each gene. A Students' t-test was used for statistical evaluation. A minimum of five samples/treatment group were used.

#### MicroRNA

The Qiagen miScript PCR system was used. For each sample (*n* = 5 per group), 1 μg of total RNA enriched with small RNA species was polyadenylated and then converted to cDNA using an oligodT primer with a universal tag and miScript Reverse Transcription mix. Real-time PCR was performed in duplicate for each sample, using a primer complementary to the universal tag and a miScript primer (Qiagen) specific for each miRNA. Amplified product was detected using SYBR Green and a CFX real-time detection system (Bio-Rad). Expression levels of miRNAs were normalized to expression levels of small nuclear RNAs RNU1A1 and RNU5A1. A Students' t-test was used to statistically evaluate the data.

### Pathway-Specific PCR Arrays

Approximately 800 ng of total RNA per sample (*n* = 6/group) was reverse transcribed using a RT^2^ first strand kit (SABiosciences™). Reverse transcription and real-time PCR reactions were carried out using RT^2^ SYBR Green PCR Master Mix on 96-well PCR arrays designed for the evaluation of mouse inflammatory cytokines and receptors (No. PAMM-011D, SABiosciences™) using a CFX real-time Detection System (BioRad). Threshold cycle values were averaged. Relative gene expression was determined according to the comparative *C*_t_ method and normalized to the *Hprt* and β-*actin* housekeeping genes. Fold changes were calculated using online PCR array data analysis software (SABiosciences™). Statistical significance was calculated using the REST method [Pfaffl et al.,^2002^].

### Pathway Analysis

Gene ontology was used to assign genes to functional categories in DAVID [Huang da et al.,^2009^]. KEGG pathways were used to identify specific biological pathways associated with the differentially expressed genes. In addition, we also conducted pathway analyses using two programs we developed to perform gene set enrichment analysis in R. KEGG gene sets were obtained using the mgug4121a.db R library. The first method is a rank-based test that takes into accounts the magnitude of the intensity value as well as the correlation between genes within a specific pathway. The advantage of this test is that no assumptions on distribution or independence are made. Genes in a pathway are ranked within each observation and the average rank is calculated for each treatment. The distance between the two treatments is calculated. Permutation analysis is used to obtain a *P*-value for each pathway (Table I in the manuscript). The second method (Design-based test) utilizes Euclidean distance between the estimated mean vector of two treatment groups as the test statistic. The null distribution of the test statistic distance is obtained using bootstrapping as described by Kerr and Churchill [2001]. The advantage of this method is that the experimental design used in the study is accounted for.

**Table I:** Gene Set Enrichment Analysis

		Probe	
		
Pathway	*P* value	aTotal	bChanging
**Rank-based test**			
Complement and coagulation cascades	0.002	123	18
Hematopoietic cell lineage	0.002	253	13
Olfactory transduction	0.003	914	2
Pantothenate and CoA biosynthesis	0.008	26	1
Systemic lupus erythematosus	0.013	222	11
Cytokine-cytokine receptor interaction	0.018	652	33
gamma-Hexachlorocyclohexane degradation	0.021	27	3
Neuro active ligand-receptor interaction	0.041	421	3
Toll-like receptor signaling pathway	0.045	332	11
**Design-based test**			
Cytokine-cytokine receptor interaction	0.001	652	33
Pantothenate and CoA biosynthesis	0.001	26	1
Hematopoietic cell lineage	0.006	253	13
Complement and coagulation cascades	0.011	123	18
Systemic lupus erythematosus	0.017	222	11
ABC transporters	0.018	87	2
Natural killer cell mediated cytotoxicity	0.018	334	13
T cell receptor signaling pathway	0.019	352	11
gamma-Hexachlorocyclohexane degradation	0.026	27	3
Fc epsilon RI signaling pathway	0.029	243	13
Asthma	0.032	87	3
Jak-STAT signaling pathway	0.032	390	10
Linoleic acid metabolism	0.041	53	6

a Total indicates the total number of known probes in a given pathway.

b Changing indicates the number of probes changing in a given pathway following the exposure.

### Preparation of Tissue Protein Extracts and Western Blotting

A random section of the frozen left lung lobe was homogenized in lysis buffer (5 M HEPES, pH 7.5, 5 M NaCl, 10% Glycerol, 1% Triton X-100, 2 M EGTA, 1 M MgCl_2_, 0.5 M NaF, 0.2 M sodium pyrophosphate, protease inhibitor cocktail tablets (Roche Applied Science)) and centrifuged. The supernatant was quantified for protein content using a Bradford protein assay reagent kit (Bio-Rad).

Total lung protein extracts were immunoblotted on 14% SDS-PAGE gels and analyzed using antibodies against Saa3 (Santa Cruz Biotechnologies). Membranes were stripped and reprobed with anti-actin antibody for normalization. Band intensities were determined by averaging the densitometric readings from three biological replicates of control and treated samples from the same gel.

### Total SAA Immunoassay

The Mouse SAA immunoassay (Invitrogen) was used to measure total Saa1 in lung tissue homogenates. The assay was conducted according to the manufacturer's instructions. Briefly, 100 μl of assay diluent and known quantities of controls (0–5 μg/ml mouse SAA supplied by the company) and individual samples (100 μg total tissue homogenates) in 50 μl of incubation buffer were loaded onto a microplate precoated with mouse Saa1 specific antibody followed by addition of biotinylated second monoclonal antibody. The plate was incubated at 37°C for 1 hr and unbound Saa1 was removed by washing. A streptavidin-HRP enzyme was then added to each well and incubated for 30 min at room temperature. Plates were washed to remove any unbound enzyme conjugate. One-hundred microliters of chromagen substrate solution was added to each well and incubated in the dark for 30 min at room temperature. The reaction was quenched by adding 100 μl of stop solution to individual wells. Optical Density for each well was determined at 450 nm using a microtiter plate spectrophotometer.

For analysis of Saa2, an Enzyme Immunoassay kit (Life Diagnostics) was used. One hundred micrograms of total protein from individual lung homogenates and controls (reference standard Saa2 0–500 ng/μl, supplied by the company) were loaded onto a plate precoated with anti-mouse Saa2 antibody along with horseradish peroxidase-conjugated polyclonal antibody. Plates were incubated for 1 hr at room temperature, wells were washed and 100 μl of chromagen was added to each well. Following incubation with chromagen for 20 min at room temperature, 100 μl of stop solution was added and the optical density was read at 450 nm in a microtiter plate reader. A minimum of 3 samples/treatment group were used.

## RESULTS

### Exposure and Particle Characterization

Mature female mice (*n* = 9/controls, 8/treated) were exposed for 1 hr per day for 11 consecutive days to 42.4 ± 2.9 (SEM) mg/m^3^ nanoTiO_2_ particles. Physicochemical characteristics of the nanoTiO_2_ used in the study are presented in detail by Hougaard et al. [2010]. Briefly, the sample consisted of a rutile TiO_2_-based material, which accounted for 70.8 wt% TiO_2_. The other major constituents were modifiers of SiO_2_ > Al_2_O_3_ > ZrO_2_ and 5.2 wt% polyalcohol coating. Mass spectrometric analyses suggested that the polyalcohol consisted of a complex mixture with 4, 6, or 8 carbon chain length. BET measurements showed that the specific surface area was 107.7 m^2^/g, which is slightly higher than what was reported by the manufacturer (70 m^2^/g). Transmission electron microscopy showed that the powder particles consisted of aggregates and agglomerates of equidimensional to needle-shaped TiO_2_ crystals with particle size ranging from less than 10 nm to more than 100 nm diameter. Rietveld analysis of powder X-ray diffractograms revealed that the average crystallite size of the TiO_2_ was 20.6 ± 0.3 nm, but an elongation was also determined.

The mice were exposed to 42.4 ± 2.9 (SEM) mg nanoTiO_2_/m^3^ particles mass concentrations as indicated by periodic filter measurements. The number of particles in the exposure atmosphere was 1.70 ± 0.20 × 10^6^/cm^3^ with major particle sizes of ∼100 nm and 4 μm. About 80% of the particles by number were between 40 and 200 nm with a maximum size of 12 μm. However, the mass–size distribution was dominated by micrometer-size particles. Sub-100 nm size particles made up less than 1% of the mass.

The concentration of TiO_2_ particles in tissues was analyzed by ICPMS and showed 38 mg TiO_2_/kg in the lungs of mice 5 days after the last exposure. The lungs of control mice did not contain TiO_2_. No TiO_2_ particles were detected in the liver tissues of the exposed or control mice [Hougaard et al.,^2010^].

### Bronchoalveolar Lavage Fluid Analysis

To assess inflammatory response to nanoTiO_2_ exposure in mouse lungs, inflammatory cell counts were performed on the bronchoalveolar lavage fluid (BALF) as described by Hougaard et al. [2010] and the response was reported in detail by Hougaard et al. [2010]. Briefly, the total number of cells in BALF from mice exposed to nanoTiO_2_ increased by 21% compared with mice exposed to air only, a change that was not statistically significant. The percentage of neutrophils increased significantly from 2% in control (air exposed) mice to 43% in the nanoTiO_2_-treated group (a 19-fold increase). However, the total number of macrophages decreased from 87% in controls to 49% in the treated group. There was a 6-fold increase in lymphocytes in the treated group and no significant changes in the total number of eosinophils and epithelial cells [Hougaard et al.,^2010^].

### Gene Expression Analysis

**Fig. 1:**
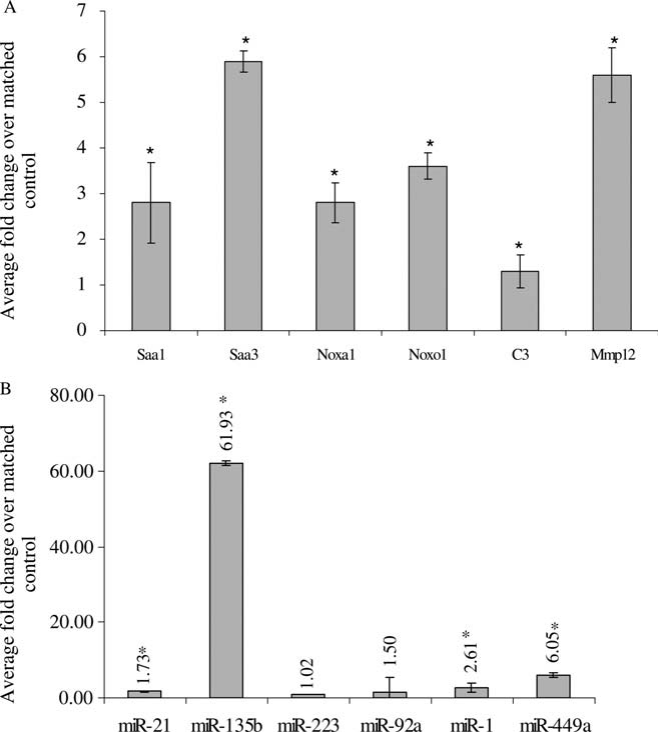
Real time quantitative PCR validation of array results. **A**: Microarray results. Data (*n* = 5 mice/group, ±SEM) are presented as average fold changes over matched controls (air exposed). Gene expression values were normalized to internal reference gene *GAPDH* and *Hprt*. **B**: MicroRNA array results. Data are presented as average fold changes over matched controls (air exposed). (*n* = 5 mice/group, ±SEM). Expression values were normalized to small nuclear RNA RNU1A1. Numbers indicate fold changes. * indicates significant by Students' t-test.

MAANOVA analysis revealed 353 transcripts that were differentially expressed (FDR—*P*-values < 0.05) compared with matched controls. Of these, 53 had fold changes >1.5 for exposed versus control (50 upregulated and 3 downregulated) (full list in Additional File 1; File 1: Supporting Information Table S1). Complete microarray data are available through the Gene Expression Omnibus at NCBI (http://www.ncbi.nlm.nih.gov/geo/), GSE 19487. A heatmap was generated using the Heatplus library [Ploner,^2008^] in R [R-Development-Core-Team,^2009^] using the log2 of the relative intensities. Any technical probe replicates were averaged using the mean. Outliers were removed based on the cluster analysis of the normalized log2 of the Cy3 and Cy5 channel. Hierarchical cluster analysis on differentially expressed genes revealed that samples within a treatment group were clustered together (Additional File 1; File 1: Supporting Information Fig. S1). Thus, a clear treatment effect was found as a result of exposure to nanoTiO_2_. The genes with increased mRNA levels included the following: *serum amyloid A-3* (*Saa3*, 4.7-fold), *chemokine (C-X-C motif) ligand 5* (*cxcl5*, 4.4-fold), *lymphocyte antigen 6 complex, locus F* (*Ly6f*, 4.3-fold), *solute carrier family 26,member 4* (*Slc26a4*, 3.4-fold), *lipocalin 2* (*Lcn2*, 3.3-fold), and *NADPH oxidase organizer 1* (*Noxo1*, 3.0-fold). Among the genes with decreased mRNA levels, the largest decrease was observed for calcium-dependent phospholipid-binding protein, *Copine5* (*Cpne5*, 2.1-fold). Real time RT-PCR confirmed the altered mRNA levels of *serum amyloid A 1*(*Saa1*), *Saa3,Noxo1,NADPH oxidase activator 1* (*Noxa1*), *matrix metallopeptidase 12* (*Mmp12*), and *complement component 3* (*C3*) in the exposed mice (Fig. 1A).

Gene ontology analysis was used to assign genes to functional categories in DAVID [Huang da et al.,^2009^]. KEGG pathways were used to identify specific biological pathways associated with the differentially expressed genes. Pathway enrichment analysis applied two programs using the mgug4121a.db R library (Table I, rank-based test [Alvo et al.,^2010^], and a design-based test). The major pathways that were identified remained the same regardless of the method applied. These included cytokine–cytokine receptor interaction, metabolism, complement and coagulation cascade, hematopoeitic cell lineage, biosynthesis of steroids, and systemic lupus erythematosus.

### Induction of Acute Phase Response Reactants

NanoTiO_2_ exposure altered the expression of several acute phase response genes (Table II) including *Saa1* and *3*. Previous work has demonstrated that acute phase response genes including *Saa* are induced in response to air pollution and inhaled particulates [Pope et al.,^2004^; Ruckerl et al.,^2006^]. Protein analysis was conducted to elucidate the role of *Saa* genes in nanoTiO_2_-induced lung response. ELISA analysis of total lung tissue homogenates could not detect Saa2 in either treatment group (data not shown). A very modest decrease (0.86-fold) in total Saa1 protein was observed in the lung tissues of exposed mice (Additional File 1; File 1: Supporting Information Fig. S2A), in contrast to the gene expression results (Table II, 2.2-fold upregulated). This observation may have been due to outlying values from two control mice (expressing high amounts of Saa1 as indicated by the large error bars, Additional File 1; File 1: Supporting Information Fig. S2A.). Alternatively, *Saa1* may be targeted post-transcriptionally by a miRNA that is also induced in response to nanoTiO_2_. Western blot analysis (no ELISA assay available) revealed a 2.2-fold increase in total Saa3 protein in the nanoTiO_2_-treated group relative to matched controls (Additional File 1; File 1: Supporting Information Fig. S2B).

**Table II:** List of all Acute Phase Response Genes Showing Fold Changes Higher Than 1.2 in exposed mice

Acute phase reactants	*P* value	Fold change^a^
**Serum amyloid A1**	**0.00**	**2.24**
**Serum amyloid A3**	**0.00**	**4.71**
**Complement protein C3**	**0.00**	**1.37**
**Complement component 1, s (C1s)**	**0.00**	**1.28**
**Complement component 3a receptor 1 (C3ar1)**	**0.00**	**1.15**
**Complement component 1, q beta polypeptide (C1qb)**	**0.00**	**1.30**
**Complement component 1, r subcomponent (C1r)**	**0.00**	**1.31**
**Complement component C1RB (C1rb)**	**0.00**	**1.21**
Fibrinogen	0.01	2.05
Coagulation factor II (F2)	0.01	1.72
Mannose binding protein	0.02	1.70
Albumin	0.01	1.79
apoA1	0.01	1.51
apoAII	0.03	1.61
alpha2-HS glycoprotein	0.00	1.85
S100A8 (calgranulin A)	0.01	−1.85
Serpina3n	0.00	1.37

Gene names in bold indicate FDR adjusted *P* value > 0.05.

a Average fold change compared with matched controls.

### Induction of Inflammatory Cytokines and Receptors

NanoTiO_2_ exposure resulted in increased mRNA levels of genes associated with cytokine–cytokine receptor signaling and chemokine signaling pathways. These include chemokine (C-X-C motif and C-C motif) ligands (*cxcl5, cxcl1 and cxcl12, ccl7, ccl9, ccl2, ccl17, ccl12, and ccl19*). We confirmed the altered mRNA levels of these genes using pathway-specific PCR arrays (mouse inflammatory cytokines and receptors; SABiosciences™) containing 70 different cytokines and chemokines. Six individual samples from the control and treatment groups were analyzed. Twenty one genes were statistically significantly differentially expressed (1.5-fold) by t-tests (data not shown) and 14 genes by the REST method [Pfaffl et al.,^2002^] (Table III and Additional File 1; File 1: Supporting Information Table S2). The analysis showed significant increase in levels of *cxcl-5* (30.0-fold), *cxcl1* (7.0-fold), *ccl2* (4.2-fold), *ccl22* (3.4-fold), *ccl7* (3.8-fold), *ccr4* (2.1-fold), and cytokine *tumor necrosis factor* (*TNF*, 1.8-fold) in lung tissues of mice treated with nanoTiO_2_. Expression of *interleukin-6,interleukin 1 beta*, and *IFN gamma* did not change. Overall, 14 transcripts that were significantly differentially expressed with FC > 1.5 as measured by the microarray were validated using the PCR arrays. Eleven of them are in agreement with the results of the microarray.

**Table III:** Results of Pathway-Specific PCR Array (Mouse Inflammatory Cytokines and Receptors)

Gene name	*P* value	Fold change^a^
Chemokine (C-X-C motif) ligand 1 (Cxcl1)	0.000	7.00
Chemokine (C-C motif) ligand 2 (Ccl2)	0.000	4.20
Chemokine (C motif) receptor 1 (Xcr1)	0.000	1.80
Chemokine (C-X-C motif) ligand 5 (Cxcl5)	0.010	30.00
Secreted phosphoprotein 1 (Spp1)	0.010	2.00
Chemokine (C-C motif) ligand 6 (Ccl6)	0.021	1.90
Complement component 3 (C3)	0.021	1.70
Chemokine (C-C motif) ligand 22 (ccl22)	0.021	3.40
Chemokine (C-C motif) receptor 4 (Ccr4)	0.021	2.20
Chemokine (C-C motif) ligand 3 (ccl3)	0.024	2.20
Chemokine (C-C motif) ligand 12 (Ccl12)	0.026	2.00
Chemokine (C-C motif) ligand 9 (Ccl9)	0.028	1.70
Tumor necrosis factor (Tnf)	0.041	1.80
Chemokine (C-C motif) ligand 7 (Ccl7)	0.048	4.00

a Average fold change compared with matched controls.

### Hepatic Gene Expression

We analyzed liver mRNA from the same mice and found differential expression of 11 genes (FDR *P*-value <0.05); however, these expression changes were modest; the largest change was a 1.6-fold downregulation of “*similar to hepatocellular carcinoma-associated gene TD26*.” The other 10 genes had fold changes below 1.2. The DNA microarray data are available through the NCBI (http://www.ncbi.nlm.nih.gov/geo/), GSE 19487. Overall ranking of all genes according to fold change demonstrated that gene expression was relatively unchanged in the livers.

### Global MiRNA Expression Changes

Agilent arrays containing 567 mouse probes were used to examine changes in miRNA following exposure to nanoTiO_2_. The data are available through the NCBI (http://www.ncbi.nlm.nih.gov/geo/), GSE 19487. Fifty-five miRNAs were significantly altered by nanoTiO_2_ exposure following FDR-adjustment (Table IV). The levels of six miRNAs were increased by more than 1.5-fold; the only downregulated miRNAs were miR-92a (1.3-fold) and miR-223 (1.4-fold)). Real time RT-PCR analysis confirmed the upregulation of miR-449a, miR-1, and miR-135b (Fig. 1B).

**Table IV:** MiRNA Results

Probe	FDR *P* value	Fold change^a^
mmu-miR-449a	0.000	2.81
mmu-miR-1	0.000	1.92
mmu-miR-135b	0.000	1.83
mmu-miR-133b	0.004	1.59
mmu-miR-144	0.008	1.53
mmu-miR-21	0.000	1.52
mmu-miR-133a	0.018	1.39
mmu-miR-34b-5p	0.000	1.33
mmu-miR-193	0.048	1.29
mmu-miR-34c	0.000	1.28
mmu-miR-141	0.001	1.28
mmu-miR-33	0.003	1.26
mmu-miR-342-3p	0.000	−1.25
mmu-miR-92a	0.007	−1.26
mmu-miR-720	0.016	−1.28
mmu-miR-223	0.001	−1.39

a Average fold change compared with matched controls.

## DISCUSSION

Short term or chronic exposure to nanoTiO_2_ particles by inhalation or instillation has revealed that nanoTiO_2_ particles induce a complex pulmonary response that potentially involves alterations in signaling cascades and associated expression of numerous genes and proteins [Lee et al.,^1985^; Bermudez et al.,^2002^; Bermudez et al.,^2004^; Warheit and Frame,^2006^]. Changes in gene expression are presumed to occur in response to exposure and in the early stages of disease development, and thus lead to the downstream biological outcome. MiRNAs are proposed to be one of the mechanisms regulating mRNA and protein levels following toxicant exposure [Hudder and Novak,^2008^; Taylor and Gant,^2008^], and thus are potentially important mediators of toxic outcome. Concurrent analysis of mRNAs and miRNAs is a powerful approach to explore the direct response of the genome to toxicant exposure.

We have recently reported the developmental and neurological effects of surface-coated nanoTiO_2_ particles [Hougaard et al.,^2010^]. Pregnant mice were exposed for 1 hr daily for 11 consecutive days to 42.4 ± 2.9 (SEM) mg/m^3^ suspended nanoTiO_2_ particles via inhalation from gestational day 8–18. The mice that failed to conceive were sacrificed 5 days following the exposure. These mice (nonpregnant adult) exhibited evidence of pulmonary inflammation by analysis of cell counts in BALF. In this study, we characterize the global mRNA and miRNA response in the lungs of the nonpregnant adult mice, to provide insight into the molecular mechanisms contributing to this effect. We also analyze global hepatic mRNA response to the inhaled nanoTiO_2_. We demonstrate alterations in pulmonary mRNA levels in pathways that are consistent with the observed and predicted effects of particle exposure and also in line with the observed phenotype. We also report perturbation of several miRNAs in the lungs of these mice. In contrast, gene expression was relatively unaffected in the livers of the same mice.

### Pulmonary Inflammation

Analysis of different cell counts in BALF revealed an increase in the total number of neutrophils, supporting the presence of pulmonary inflammation, 5 days following nanoTiO_2_ exposure in mature female mice from this study [Hougaard et al.,^2010^]. Long-term pulmonary inflammation following inhalation of nanoTiO_2_ has previously been documented in both mouse and rat models [Bermudez et al.,^2004^; Ma-Hock et al.,^2009^; van Ravenzwaay et al.,^2009^]. For example, studies have demonstrated increased infiltration of neutrophils 2 weeks following inhalation of 100 mg/m^3^ mixed anatase and rutile nanoTiO_2_ for 6 hrs/day for 5 days [Ma-Hock et al.,^2009^; van Ravenzwaay et al.,^2009^]. Similarly, mice exposed to 10 mg/m^3^ nanoTiO_2_ for 6 hrs/day, 5 days/week for 13 weeks showed persistent increases in the number of neutrophils following a 52 day recovery period [Bermudez et al.,^2004^]. These studies, along with the data presented here, suggest that nanoTiO_2_-induced pulmonary inflammation is characterized by neutrophil infiltration.

This study also supports previous work demonstrating that surface-treated nanoTiO_2_ particles are inflammogenic. For example, intratracheal instillation of surface-treated (alumina or alumina and amorphous silica) nanoTiO_2_ caused pulmonary inflammation in rats [Warheit et al.,^2005^]. High doses of silanized hydrophobic ultrafine TiO_2_ instilled into rats resulted in increased lung toxicity [Pott et al.,^1998^]. Mild inflammatory changes were noted in rats instilled with low doses of silanized nanoTiO_2_ [Rehn et al.,^2003^]. Similar inflammogenic responses were observed in several other studies using ultrafine nonsurface-modifed nanoTiO_2_ both *in vitro* and *in vivo* [Baggs et al.,^1997^; Drumm et al.,^1999^; Bermudez et al.,^2002^; Bermudez et al.,^2004^; Renwick et al.,^2004^; de Haar et al.,^2006^]. The extent of inflammation observed in this study is in line with the overall calculated mass of nanoTiO_2_ deposited in the pulmonary region (72.5 μg with a surface area of 77.6 cm^2^). Differential cell count analysis of BALF revealed that 43% of the cells were neutrophils [Hougaard et al.,^2010^]. This finding is in close agreement with a previous experiment where mice exposed by instillation to nanoTiO_2_ with a surface area of 50 cm^2^ exhibited 45% neutrophils in BALF [Oberdorster et al.,2005b]. A linear correlation between the total number of neutrophils in BALF and particle surface area was noted in mice exposed for 24 hrs to five different types of nanoparticles, including the nanoTiO_2_ studied in this study (Saber et al., personal communication). Similar relationships between surface area and total number of neutrophils has also been reported by Tran et al. [Tran et al.,^2000^]. Taken together, these studies demonstrate that nanoTiO_2_ exposure promotes proinflammatory activities across a wide range of doses, necessitating an understanding of the molecular pathways involved in this response.

### mRNA Expression Profiling

Global transcriptional profiling of lung samples from adult female mice exposed to nanoTiO_2_ revealed significant differential expression of several inflammation modulators. Inhalation exposure to 42.4 ± 2.9 (SEM) mg/m^3^ nanoTiO_2_ for 1 hr every day for 11 consecutive days induced significant changes in the mRNA levels of several chemokine genes. These include *ccl2* (*MCP-1 alpha*), *cxcl1* (*keratinocyte cell-derived chemokine, KC*), *cxcl5* (*epithelial cell-derived neutrophil activating peptide ENA78*), *ccl22* (*macrophage-derived chemokine*), *ccl7* (*monocyte chemotactic protein 3, MCP3*), *ccl9/MIP1 gamma*, and *ccl3* (*monocyte inflammatory protein, MIP-1 alpha*). These genes participate in the modulation of chemotaxis, infiltration of neutrophils, and epithelial proliferation. Because it is difficult to interpret the biological relevance of small fold changes of individual genes, we emphasize changes in pathways and groups of genes (i.e., pathways, and Gene Ontology molecular functions and biological processes) in our discussion below. When many genes that interact together to carry out a biological function are perturbed, this is more likely to be biologically relevant than an individual gene within that family. Thus, the gene expression changes we observed collectively provide molecular insight into the findings on inflammation described earlier.

Neutrophil sequesteration is a critical event during pulmonary inflammation and involves multiple steps including the following: Increased production of chemokines and cell adhesion molecules, and enhanced interactions between cells. Cxcl1, macrophage inflammatory protein −2 (MIP2), and cxcl5 are the major chemokines for neutrophil recruitment and accumulation in mouse lungs. Cxcl1 and MIP2 are produced predominantly by the infiltrating myeloid cells (macrophages and neutrophils) and increases in their levels in response to a stimulant are rapid and transient. In contrast, cxcl5 is produced by resident cells, such as alveolar epithelial type II cells. Elevated levels of cxcl5 persist for days during lung inflammation. Preferential and persistent induction of cxcl5 in resident cells may be important in establishing cellular communication between myeloid cells and resident cells, which is required for initiation, maintenance and resolution of an inflammatory process [Jeyaseelan et al.,^2005^]. We observed high levels of *cxcl5* 5 days following the last exposure in this study. This finding indicates epithelial cell activation and may suggest continued lung injury and persistent pulmonary inflammation.

Increased expression of ccl3, cxcl5, and cxcl1 has been shown following nanoTiO_2_ exposure in several *in vivo* mouse models; these findings were associated with the development of pulmonary inflammation, granulomas, emphysema, and pulmonary host defense [Driscoll et al.,^1993^; Park et al.,^2010^; Rossi et al.,^2010^]. Disregulation of inflammatory pathways following inhalation of nanoTiO_2_ has also been shown by Chen et al. using a DNA microarray approach [Chen et al.,^2006^]. These authors used small (∼6,000 clones) “home-made” cDNA microarrays to show that a single intratracheal instillation of 0.1 mg nanoTiO_2_ (rutile, uncoated pure particles of 19–21 nm in size) caused severe pulmonary inflammation with alterations in genes involved in several pathways that overlap with this work (chemokines and coagulation complement cascade). Chemotaxis and immune response pathways, and Th1 and 2 type cytokines were also differentially regulated in the BALF of mice treated with a single intratracheal dose of 20 mg/kg nanoTiO_2_ (P25, primary particle size 20 nm) 14 days after the last exposure [Park et al.,^2009^]. In many of the aforementioned studies, comparatively high doses of particles were directly deposited into the lungs of the animals by intratracheal instillation. In this study, mice were exposed using a biologically relevant exposure route to a relatively low dose of nanoTiO_2_ (mimicking the occupational scenario in terms of the total mass administered) and similar changes in gene expression profiles were noted despite differences in doses and time points. In contrast to our data and the studies described earlier, Rehn et al. [2003] reported no signs of inflammation in rats exposed to a range of occupationally relevant low doses of surface-treated nanoTiO_2_. Discrepancies between studies may be due to the targeted analysis of specific markers of inflammation and endpoints (e.g., total protein and BAL cells) applied by Rehn et al. [2003] compared with analysis of the entire transcriptome. Many nanotoxicological studies obtain data from a single endpoint representative of a particular mechanism of action, focusing on specific sets of genes or even a single pathway. The drawback of this approach is that the mechanism of action must be known beforehand; moreover, the expected phenotype or the physiological change may not be apparent in the very low dose range. Global gene expression profiles provide a snapshot of multiple cellular processes operating in a single experiment without prior knowledge of the mechanism of action. It is particularly suitable to identifying early molecular changes predictive of eventual pathological outcome. Thus, we demonstrate that global gene expression signatures support the observed phenotypes for exposure to nanoTiO_2_ that response can be measured in the low-dose range, and the results obtained can be used to provide insight into the molecular mechanisms operating in response.

Pulmonary response to nanoTiO_2_ also causes increase in levels of genes in the acute phase response pathway (Table II) including *Saa1, Saa3, C3*, complement components *1s* (*C1s*), *3ar1* (*C3ar1*), *1qb* (*C1qb*), *1r* (*C1r*), and *1rb* (*C1rb*). Moreover, several other acute phase proteins exhibited fold changes >1.5-fold with unadjusted *P*-values < 0.05. These include *C- reactive protein,fibrinogen,coagulation factor II,mannose binding protein,albumin,apoAI,apoAII*, and *a2-hs-glycoprotein* (regulator of SAA) (Table II). The complement cascade includes 30 proteins (some of which are enzymes), cofactors, inhibitors, and membrane-associated proteins. These molecules act in host defense by promoting phagocytosis and inflammation, which can activate the complement cascade resulting in chemotaxis and pulmonary inflammation. Acute-phase proteins have been used as biochemical markers of disease for numerous inflammatory processes. Induction of acute phase response has previously been observed in rodents following particle exposure. *Saa* is a generic name for a family of apolipoproteins [Meek and Benditt,^1986^] synthesized in response to activated monocyte/macrophage-released cytokines such as IL-1 and IL-6 [Ganapathi et al.,^1988^; Jiang et al.,^1995^; Jensen and Whitehead,^1998^]. Although the liver is the major site of synthesis of *Saa* [Morrow et al.,^1981^; Lowell et al.,^1986^], extrahepatic tissues including lung, spleen, adrenal gland, and others have been shown to express varying levels of individual members of *Saa* genes in response to different inflammatory stimuli [Meek and Benditt,^1986^]. In this study, *Saa* genes (*1* and *3*) were strongly induced in the lungs of mice exposed to nanoTiO_2_. The increase in *Saa3* mRNA levels was much greater (5.9-fold) than *Saa1* (2.8-fold). Corresponding protein levels of Saa3 were also changed in lung tissue homogenates of the treated group (Supporting Information Fig. S2B) but no change was observed for Saa1. Previous work has demonstrated that *Saa* genes are induced in mouse lung in response to dust and lipopolysaccharide (LPS) [Meek and Benditt,^1986^; Andre et al.,^2006^; Park et al.,^2010^]. In keeping with this study, while injection of LPS resulted in increased mRNA levels of all three *Saa* genes in liver, *Saa3* was predominantly expressed in lungs. Similarly, the largest increase in *Saa3* mRNA levels was noted in mouse lungs after ultrafine particle exposure [Andre et al.,^2006^].

Select upregulation of Saa3 and associated proteins, together with increased gene expression of a suite of cytokines, chemokines, and metalloproteases in our model suggests a critical role for Saa3 in nanoTiO_2_-induced pulmonary inflammation/immune response. In premetastatic lungs, Saa3 induction by chemoattractants such as S100A8 plays a role in myeloid cell accumulation and acts as a positive feedback regulator for chemoattractant secretion [Hiratsuka et al.,^2008^]. In this study, *S100A8* mRNA levels were decreased compared with the controls (−1.9-fold; unadjusted *P*-value < 0.05) in the treatment group suggesting the involvement of a different regulatory mechanism for Saa3. Although the cytokines IL-1, IL-6, and TNF play a role in the expression of *Saa* genes, only *TNF* was marginally upregulated in our model. We also observed a marginal increase in TNF protein in BAL cells as well in lung tissue homogenates by protein arrays (data not shown), suggesting a possible role for TNF in *Saa3* expression in response to nanoTiO_2_.

Several chemokines (*cxcl5, cxcl1*, and *ccl3*) and components of the acute phase pathway (*Saa3, c3*) measured in this study are rapidly induced in response to acute injury or stress. However, the increase in their expression levels is transient and falls rapidly once the stimulus is removed or the injury is repaired. Thus, high and persisting levels of these acute stressors 5 days after the last exposure may reflect the magnitude of the injury and indicate initiation of secondary pathological conditions.

In addition to confirming the response of these pathways, we also found a dramatic increase in *polymeric Ig receptor* (*PIgr*, 2.0-fold), *solute carrier family 26, member 4* (*Slc26a4*, 3.4-fold), and *lipocalin 2* (*Lcn2*, 3.3-fold). These genes are involved in mucosal immune defense. *NADPH oxidase organizer 1* (*Noxo1*), a gene associated with antimicrobial defense [Flo et al.,^2004^; Schneeman et al.,^2005^; Nakao et al.,^2008^], was also upregulated by 3-fold. We also found upregulation of the proinflammatory genes *Vanin-1* and *3* (*Vnn-1,−3*, 2.6- and 1.9-fold, respectively) in nanoTiO_2_-exposed lungs [Nitto et al.,^2008^]. Thus, our results confirm strong activation of pulmonary immune and inflammation in response to nanoTiO_2_ inhalation.

Despite significant upregulation of several genes associated with acute phase, inflammation and immune response in lung, the hepatic transcriptome of these mice was unaffected. These findings agree with our previous report showing few changes in hepatic transcription in mice exposed by inhalation to doses of carbon black and diesel exhaust that caused substantial pulmonary inflammation [Saber et al.,^2009^]. The results suggest that systemic effects in liver are secondary to pulmonary inflammation and may not require gene expression.

### NanoTiO_2_ Exposure

Based on the exposure conditions, each animal in this study was exposed to ∼840 μg of nanoTiO_2_ (11 days × 1 hr/day × 40 mg/m^3^ × 0.0018 m^3^ inhaled/hr = 0.840 mg/animal). It is predicted that the majority of nanosized particles deposit in the lung once inhaled. The deposition of particles in the lungs depends on the size of the particles. In this study, the mass of the airborne fraction contained predominantly micrometer-sized particles. On the basis of the deposition model described by Jacobsen et al. [2009], we expected a deposition of 72.5 μg in the pulmonary region, 48 μg in the tracheobronchial region, 356 μg in the gastrointestinal tract, and 267 μg of nanoTiO_2_ in the skull. Hence, with an average lung weight of 274 mg, the estimated deposited pulmonary dose amounts to 265–439 mg/kg nanoTiO_2_ corresponding to 112–159 mg nanotitanium (Ti)/kg in the lung, depending on whether pulmonary or bronchopulmonary regions are considered. Detection in lung tissue using ICPMS revealed 38 mg/kg Ti in the lungs [Hougaard et al.,^2010^], indicating that although significant clearing of the lungs had taken place during the 16 days after the first exposure, considerable amounts of nanoTiO_2_ were still present 5 days postexposure. This corresponds to 24–34% of predicted nanoTiO_2_ deposition in lungs. Our results are in agreement with Rossi et al. [2010], who exposed mice by inhalation to 10 mg/m^3^ of surface-coated nanoTiO_2_ (40 nm) for a total of 32 hrs. They showed ∼60% of the deposited nanoTiO_2_ was cleared over a 4-week period [Rossi et al.,^2010^]. This suggests that the small particle size leads to alveolar deposition with subsequent slow clearing [Oberdorster,^1995^]. Depending on the particle size, some of the inhaled nanoTiO_2_ is also expected to be deposited in the nasal region. Approximately 90% of 1 nm, 20–30% of 5–10 nm, and less than 10% of 20 nm particles deposited in the nasopharyngeal region. A small fraction of the total particle mass deposited in the nasal region has previously been shown to be efficiently translocated to olfactory bulb in brain through axonal transport via olfactory neurons [Oberdorster et al.,^2002^,^2004^]. However, particles deposited in the nasal region of the respiratory system would be quickly cleared either by blowing the nose, sneezing, or by efficient mucociliary transport mechanism. As such, we have not assessed the deposition of particles in the upper respiratory tract and hence their uptake by the central nervous system. Since particles in the nasal region are rapidly cleared, and since we have measured the gene expression changes 5 days after the last exposure, we believe that the contribution of nasally deposited particles to overall outcome in the present model is likely to be minimal.

The dose administered in this study corresponds to the highest occupational exposure limit for dust (in terms of total mass administered, 5 mg/m^3^ during an 8 hr working day) in Denmark. Thus, this study used a relatively low, occupationally relevant exposure regime in combination with robust and conservative statistical analysis of a sufficiently large sample size of animals. However, one could argue that considering the slow clearance of the particles (as observed in this study), along with the dose used, lung overburden has occurred. Although the dose rate was high, the deposited dose is only a fraction of what would result in overload (i.e., slower motility of alveolar macrophages and impaired mucociliary clearance). Moreover, the particles in this study were surface coated with Si, Zr, and Al. It has been shown that surface-coated nanoTiO_2_ are more toxic than benign pure particles of nanoTiO_2_ [Rossi et al.,^2010^]. Although we did not test the possibility that surface coatings (Si, Al, and Zr) themselves contributed to the toxicity, it is documented in the literature that Si, Zr, and Al are less toxic when administered on their own [Rossi et al.,^2010^], suggesting that the slower clearance and retention of smaller size particles in the lung tissue could be the reason behind observed pulmonary response. Here, we confirm that nanoTiO_2_ inhalation results in changes in proinflammatory, immune response and complement cascade-related genes that persist for relatively long periods of time after the final exposure. The observed fold changes for any particular gene in our study were much lower than the other reports, which may be due to the lower doses used, the differences in exposure set up, or the types of particles used. However, a large portion of these changes were validated with quantitative RT-PCR and RT-PCR arrays. Thus, we argue that the pulmonary response observed in our study is likely due to the highly toxic nature of the particles and not lung overburden.

Although the mice in this study were exposed to doses near the occupational exposure limit for dust in Denmark in terms of total mass administered, it might be argued that the dose is high if the hours of exposure are considered (i.e., delivered in 1 hr vs. exposure over an 8 hr work day). Genomic tools have not been extensively used to understand nanoassociated toxicity. Using animals that exhibit an inflammatory response following the exposure, this work was initiated to establish proof of principle that genomic tools can be applied to characterize the effects of nanoparticles at relatively low doses and reveal the molecular perturbations that persist at this time point that contribute to the observed phenotype. However, the observed overall response at the transcriptional level must be investigated in studies incorporating multiple doses, time points, and different types of nanoTiO_2_, to truly dissect the mechanism of acute exposure and distinguish between immediate early effects and persistent effects. Thus, multiple time points and doses were not conducted in this study, but will be the subject of future work.

### MiRNA Expression Profiling

Despite a dramatic increase in *Saa1* gene transcript levels, we did not see a correlated increase in its protein synthesis. Similarly, in contrast to the ∼5-fold transcriptional increase of *Saa3* mRNA, Saa3 protein was increased by only 2-fold. One mechanism that controls translation of proteins involves targeted translational repression by miRNAs. Thus, to address the observed discrepancies between the transcription and protein expression profiles and to explore the potential role of miRNAs in mitigating mRNA and translation in response to particle exposure, we analyzed global changes in pulmonary miRNA expression using DNA microarrays. Total RNA from lung tissues of control and exposed mice was used. The analysis revealed 55 statistically significant changes; 16 were altered by more than 1.2-fold and six had fold changes >1.5 (Table IV). Because an individual miRNA may target hundreds of mRNAs, a small change in a miRNA may have significant repercussions on transcription and translation. As such, we explored changes both above and below the 1.5-fold threshold.

Some of the miRNAs that are altered in response to nanoTiO_2_ have been implicated in inflammation and immune response. For example, upregulation of miR-21 and downregulation of miR-1 has recently been observed in IL-13 transgenic mice with allergic airway inflammation [Lu et al.,^2009^]. MiR-21 is expressed by inflammatory leukocytes in asthmatic lung [Jin et al.,^2008^], hematopoietic cells [Landgraf et al.,^2007^], and in macrophages and dendritic cells [Lu et al.,^2009^]. MiR-21 is induced in murine macrophages following LPS challenge [Lu et al.,^2009^], all suggesting a critical association for miR-21 in inflammation and immune response. MiR-1 levels increase during myocardial differentiation of mouse embryonic stem cells [Takaya et al.,^2009^]. Farraj et al. [2010] have shown that miR-1 and miR-21 are significantly downregulated in the myocardium of rats exposed to synthetic particulate matter. Other miRNAs affected by nanoTiO_2_ exposure have been linked to a cancer phenotype. For example, differential expression of miR-135b is found in human colon cancer samples [Sarver et al.,^2009^] and miR-449a is associated with antitumor activity in prostate cancer cells [Noonan et al.,^2009^].

Analysis by qRT-PCR confirmed the higher levels of miR-449a (fold six), miR-1 (2.6-fold), and miR-135b (60-fold) in this study (Fig. 1B) but not the suppression of miR-223 and miR-92a. Analysis of curated targets in TarBase (http://diana.cslab.ece.ntua.gr/tarbase/) did not reveal any known targets of miR-449a or miR-135b. As such, we searched for mRNA targets of miR-135b and miR-449a using the target-prediction softwares TargetScan and Pictar. Predicted targets for miR-135b (TargetScan or Pictar) that were differentially expressed (downregulated) included the following: *checkpoint suppressor1,runt related transcription factor 2* (*runx2*), and *phosphodiesterase 8b*. Predicted targets of miR-449a that were differentially expressed included the following: *lymphoid enhancer-binding factor 1* (*lef1*) and *Kit ligand*. *Runx2* and *lef1* are the downstream targets of WNT signaling suggesting that miR-135b and miR-449a may have a common function in regulating WNT signaling. However, it appears that the effect of the observed 6- and 60-fold induction of miR-449a and miR-135b on transcript levels of their predicted targets is negligible. There may be several reasons to explain the lack of changes in target mRNAs. First, the genes targeted by miR-449a and miR-135b in response to particle exposure may be different than the ones predicted *in silico*. Second, the majority of the genes changing in response to nanoTiO_2_ in our model are acutely induced and therefore the magnitude of their induction is expected to be 10–100-fold higher immediately following the exposure. Therefore, even if some of these genes are directly targeted by the miRNAs at the transcriptional level, their suppression may not be below those of control levels. Third, it is also possible that the predominant mode of action for these miRNAs is through translational repression.

Currently, the causes and consequences of miRNA response following exposure to nanoTiO_2_ are unknown. However, changes at the transcriptional level of several target genes are implicated in acute phase and inflammatory response. Thus, miR-1, miR-135b, and miR-449a may play a role in these biological processes. We have recently confirmed induction of miR-135b in other inflammatory models including particle-induced inflammation (Halappanavar et al., unpublished data). The induction of several inflammatory chemokines and acute phase components is well characterized in response to particle exposure, including nanoTiO_2_ (as described in detail earlier). However, the possibility that these molecular pathways could be under the direct control of miRNAs that play a role in particle-induced response is yet to be demonstrated. We hypothesize that some of the genes, including the *Saa* family and chemokines, are the direct targets of miR-135b and that miR-135b could be acting to resolve the inflammation process. A similar hypothesis was also proposed by Jardim et al. [2009]. These authors suggest that disruption in miRNA expression in response to diesel exhaust particles (DEP) in human airway epithelial cells could be implicated in modulation of the inflammatory process induced by DEP. Further characterization of miR-135b responsive genes and their role in particle-induced pulmonary response is being carried out in our laboratory to determine the biological relevance of these changes.

The miRNA field is still developing in parallel with more powerful tools to study their expression and activity. At present, a number of published studies describe changes in the expression of several miRNAs in diseases (reviewed in [Sun and Tsao,^2008^]) or in certain biological processes *in vitro* and *in vivo* [Bueno et al.,^2008^; Lynam-Lennon et al.,^2009^]. However, there are very few studies that have characterized changing mRNA and miRNA profiles in target tissues *in vivo* in response to toxic substances (reviewed in [Lema and Cunningham,^2010^]). To our knowledge, this is the first report on particle-induced pulmonary miRNA changes. Integration of gene and miRNA expression profiles with reports examining multiple endpoints at the molecular, cellular, tissue, and physiological levels will be very useful in understanding the toxicity of particles in the context of the whole organism. Further studies incorporating acute, subchronic, and chronic doses with different types of nano TiO_2_ particles and multiple time points are needed to validate our findings.

## CONCLUSIONS

This work demonstrates the induction of acute phase reactants, chemoattractants, immune and host defense genes following exposure to occupationally relevant levels of surface-coated titanium dioxide nanoparticles via a biologically relevant exposure route. These findings are in keeping with the expected health outcome of chronic nanoparticle exposure, and the changes persist for up to 5 days following exposure. In parallel with perturbation of these genes, specific miRNAs are also induced in the lungs in response to nanoTiO_2_. The role of miRNAs in response to particle-induced lung injury is now under investigation in our laboratory.

*Competing interests:* There are no financial competing interests to disclose.
