# Supplementary material for: Pulmonary Response to Surface-Coated Nanotitanium Dioxide Particles Includes Induction of Acute Phase Response Genes, Inflammatory Cascades, and Changes in MicroRNAs: A Toxicogenomic Study
Source: Environ Mol Mutagen. 2011 Jan 21;52(6):425–39. doi: 10.1002/em.20639 (PMC3210826; doi:10.1002/em.20639)
Supplement: Supplementary file 1 [file em0052-0425-SD1.doc]

**Figure S1**

Hierarchical cluster analysis of all samples. Heat map represents all genes (353) that are statistically significantly differentially expressing. Green bars represent high expression levels, red represent low expression levels and black bars are similar to the normalized median gene expression values. The numbers refer to individual samples (mice), C: Controls, E: NanoTiO_2_


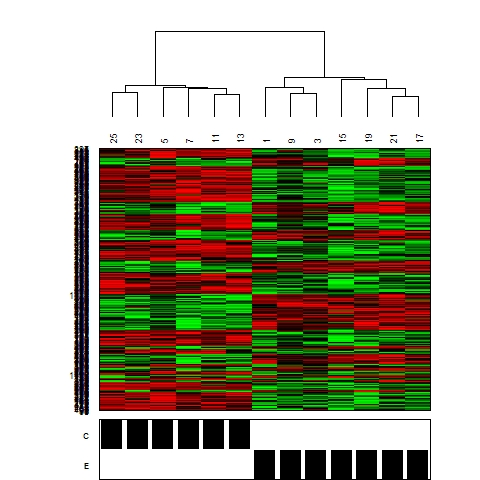


**Figure S2A**

Total immunoreactive Saa1 in 30 ugs of total lung tissue extracts. Data represent relative levels of Saa1 in 30 ugs of total lung protein analysed (n = 5, ± SEM).

**Figure S2B**

Quantification of Western blot for Saa3 protein in lung tissue extracts. Data are presented as relative amount (arbitrary units) of total Saa3 protein in control and treated samples (n = 3 mice/group, ± SEM). *indicates statistically significant by Student’s T-test.

| **Table S1 Statistically significant, differentially expressing genes (p value < 0.05)** | |  |  |
| --- | --- | --- | --- |
|  |  |  |  |
| **Genbank** | **Description** | **p-value** | ***FC** |
| NM_011315 | serum amyloid A 3 (Saa3) | 0.0000 | 4.71 |
| NM_009141 | chemokine (C-X-C motif) ligand 5 (Cxcl5) | 0.0000 | 4.39 |
| NM_008530 | lymphocyte antigen 6 complex, locus F (Ly6f) | 0.0000 | 4.30 |
| NM_011867 | solute carrier family 26, member 4 (Slc26a4) | 0.0000 | 3.41 |
| NM_008491 | lipocalin 2 (Lcn2) | 0.0000 | 3.30 |
| NM_027988 | NADPH oxidase organizer 1 (Noxo1) | 0.0000 | 2.99 |
| NM_011704 | vanin 1 (Vnn1) | 0.0000 | 2.55 |
| NM_009117 | serum amyloid A 1 (Saa1) | 0.0000 | 2.24 |
| AK005011 | adult male liver cDNA, RIKEN full-length enriched library, | 0.0000 | 2.15 |
| NM_153166 | copine V (Cpne5) | 0.0000 | -2.14 |
| NM_009890 | cholesterol 25-hydroxylase (Ch25h) | 0.0000 | 2.11 |
| NM_013654 | chemokine (C-C motif) ligand 7 (Ccl7) | 0.0000 | 2.08 |
| NM_011082 | polymeric immunoglobulin receptor (Pigr) | 0.0000 | 2.04 |
| NM_013654 | chemokine (C-C motif) ligand 7 (Ccl7) | 0.0000 | 2.01 |
| NM_139142 | X transporter protein 3 similar 1 gene (Xtrp3s1) | 0.0000 | 1.98 |
| NM_011338 | chemokine (C-C motif) ligand 9 (Ccl9) | 0.0000 | 1.97 |
| NM_009969 | colony stimulating factor 2 (granulocyte-macrophage) (Csf2) | 0.0000 | 1.93 |
| NM_011333 | chemokine (C-C motif) ligand 2 (Ccl2) | 0.0000 | 1.92 |
| NM_007802 | cathepsin K (Ctsk) | 0.0000 | 1.91 |
| NM_011979 | vanin 3 (Vnn3) | 0.0000 | 1.84 |
| NM_011332 | chemokine (C-C motif) ligand 17 (Ccl17) | 0.0000 | 1.84 |
| NM_008176 | chemokine (C-X-C motif) ligand 1 (Cxcl1) | 0.0000 | 1.78 |
| AK122570 | mRNA for mKIAA1916 protein | 0.0000 | 1.77 |
| NM_031254 | triggering receptor expressed on myeloid cells 2 (Trem2) | 0.0000 | 1.77 |
| NM_183249 | RIKEN cDNA 1100001G20 gene | 0.0000 | 1.70 |
| NM_011593 | tissue inhibitor of metalloproteinase 1 (Timp1) | 0.0000 | 1.70 |
| NM_007493 | asialoglycoprotein receptor 2 (Asgr2) | 0.0000 | 1.70 |
| A_52_P517668 | Unknown | 0.0000 | 1.67 |
| NM_011267 | regulator of G-protein signaling 16 (Rgs16) | 0.0000 | 1.63 |
| NM_001038604 | C-type lectin domain family 5, member a (Clec5a), transcript variant 1 | 0.0000 | 1.63 |
| NM_009263 | secreted phosphoprotein 1 (Spp1) | 0.0000 | 1.62 |
| NM_033075 | DNA segment, Chr 17, human D6S56E 5 (D17H6S56E-5) | 0.0000 | 1.60 |
| NM_145581 | sialic acid binding Ig-like lectin F (Siglecf) | 0.0000 | -1.59 |
| NM_172204 | NADPH oxidase activator 1 (Noxa1) | 0.0000 | 1.58 |
| NM_027836 | membrane-spanning 4-domains, subfamily A, member 7 (Ms4a7), transcript variant 1 | 0.0000 | 1.58 |
| NM_010215 | interleukin 4 induced 1 (Il4i1) | 0.0000 | 1.56 |
| AK144269 | 11 days embryo spinal cord cDNA, RIKEN full-length enriched library, | 0.0000 | 1.56 |
| NM_027836 | membrane-spanning 4-domains, subfamily A, member 7 (Ms4a7), transcript variant 1 | 0.0000 | 1.55 |
| NM_011331 | chemokine (C-C motif) ligand 12 (Ccl12) | 0.0000 | 1.55 |
| NM_054098 | STEAP family member 4 (Steap4) | 0.0000 | 1.55 |
| NM_008605 | matrix metallopeptidase 12 (Mmp12) | 0.0000 | 1.55 |
| NM_008399 | integrin, alpha E, epithelial-associated (Itgae), transcript variant 1 | 0.0000 | 1.54 |
| NM_033075 | DNA segment, Chr 17, human D6S56E 5 (D17H6S56E-5) | 0.0000 | 1.53 |
| NM_001008533 | adenosine A1 receptor (Adora1), transcript variant 1 | 0.0000 | 1.51 |
| NM_007431 | alkaline phosphatase 2, liver (Akp2) | 0.0000 | 1.51 |
| NM_028788 | RIKEN cDNA 1300002K09 gene (1300002K09Rik) | 0.0000 | 1.50 |
| NM_015811 | regulator of G-protein signaling 1 (Rgs1) | 0.0000 | 1.50 |
| AJ426449 | mRNA for Spink5 protein | 0.0000 | 1.48 |
| NM_009778 | complement component 3 (C3) | 0.0000 | 1.48 |
| NM_019707 | cadherin 13 (Cdh13) | 0.0000 | -1.48 |
| NM_010766 | macrophage receptor with collagenous structure (Marco) | 0.0000 | 1.48 |
| NM_008223 | serine (or cysteine) peptidase inhibitor, clade D, member 1 (Serpind1) | 0.0000 | 1.45 |
| NM_018746 | inter alpha-trypsin inhibitor, heavy chain 4 (Itih4) | 0.0000 | 1.44 |
| NM_009841 | CD14 antigen (Cd14) | 0.0000 | 1.43 |
| NM_144945 | leucine-rich repeat LGI family, member 2 (Lgi2) | 0.0000 | 1.43 |
| ENSMUST00000035300 | AGENCOURT_10813779 NIH_MGC_156 cDNA clone IMAGE:6758910 5' | 0.0000 | -1.42 |
| NM_021050 | cystic fibrosis transmembrane conductance regulator homolog (Cftr) | 0.0000 | 1.42 |
| NM_007894 | eosinophil-associated, ribonuclease A family, member 1 (Ear1) | 0.0000 | -1.42 |
| NM_009856 | CD83 antigen (Cd83) | 0.0000 | 1.41 |
| NM_001029929 | zinc finger, MYND-type containing 15 (Zmynd15) | 0.0000 | 1.41 |
| NM_011659 | tumor necrosis factor receptor superfamily, member 4 (Tnfrsf4) | 0.0000 | 1.41 |
| AK042173 | 3 days neonate thymus cDNA, RIKEN full-length enriched library, | 0.0000 | -1.39 |
| BC053041 | low density lipoprotein receptor, mRNA | 0.0000 | 1.39 |
| NM_031195 | macrophage scavenger receptor 1 (Msr1) | 0.0000 | 1.38 |
| NM_011888 | chemokine (C-C motif) ligand 19 (Ccl19) | 0.0000 | 1.38 |
| NM_053110 | glycoprotein (transmembrane) nmb (Gpnmb) | 0.0000 | 1.38 |
| NM_009705 | arginase type II (Arg2) | 0.0000 | 1.37 |
| NM_009252 | serine (or cysteine) peptidase inhibitor, clade A, member 3N (Serpina3n) | 0.0000 | 1.37 |
| NM_007914 | ets homologous factor (Ehf) | 0.0000 | 1.35 |
| NM_020498 | lymphocyte antigen 6 complex, locus I (Ly6i) | 0.0000 | 1.35 |
| NM_013532 | leukocyte immunoglobulin-like receptor, subfamily B, member 4 (Lilrb4) | 0.0000 | 1.34 |
| NM_011612 | tumor necrosis factor receptor superfamily, member 9 (Tnfrsf9) | 0.0000 | 1.33 |
| NM_177960 | isopentenyl-diphosphate delta isomerase (Idi1), transcript variant 2 | 0.0000 | 1.33 |
| NM_025436 | sterol-C4-methyl oxidase-like (Sc4mol) | 0.0000 | 1.33 |
| NM_134469 | farnesyl diphosphate synthetase (Fdps) | 0.0000 | 1.32 |
| NM_153166 | copine V (Cpne5) | 0.0000 | -1.32 |
| NM_053112 | eosinophil-associated, ribonuclease A family, member 10 (Ear10) | 0.0000 | -1.32 |
| NM_030707 | macrophage scavenger receptor 2 (Msr2) | 0.0000 | 1.32 |
| BC066035 | zinc finger, RAN-binding domain containing 3, mRNA | 0.0000 | 1.31 |
| NM_023143 | complement component 1, r subcomponent (C1r) | 0.0000 | 1.30 |
| NM_008610 | matrix metallopeptidase 2 (Mmp2) | 0.0000 | 1.30 |
| NM_007423 | alpha fetoprotein (Afp) | 0.0000 | -1.30 |
| NM_130861 | solute carrier organic anion transporter family, member 1a5 (Slco1a5) | 0.0000 | 1.28 |
| NM_001002786 | RIKEN cDNA 9830134C10 gene (9830134C10Rik) | 0.0000 | -1.28 |
| NM_138313 | Bcl2 modifying factor (Bmf) | 0.0000 | 1.28 |
| XM_985599 | PREDICTED: RIKEN cDNA 2210011C24 gene (2210011C24Rik) | 0.0000 | -1.27 |
| NM_144938 | complement component 1, s subcomponent (C1s) | 0.0000 | 1.27 |
| CJ042244 | CJ042244 RIKEN full-length enriched mouse cDNA library | 0.0000 | 1.27 |
| NM_001039050 | protein kinase inhibitor beta, cAMP dependent, testis specific (Pkib), | 0.0000 | 1.26 |
| NM_020010 | cytochrome P450, family 51 (Cyp51) | 0.0000 | 1.24 |
| BC091652 | fibroblast growth factor receptor 2, mRNA | 0.0000 | 1.24 |
| NM_023065 | interferon gamma inducible protein 30 (Ifi30) | 0.0000 | 1.24 |
| NM_174850 | RIKEN cDNA A930021H16 gene (A930021H16Rik) | 0.0000 | 1.23 |
| NM_009657 | aldolase 3, C isoform (Aldoc) | 0.0000 | 1.21 |
| NM_172814 | low density lipoprotein-related protein 12 (Lrp12) | 0.0000 | 1.21 |
| M20632 | Mouse LLRep3 protein mRNA from a repetitive element, complete cds | 0.0000 | 1.20 |
| NM_008433 | potassium intermediate/small conductance calcium-activated channel, (Kcnn4) | 0.0000 | 1.20 |
| NM_011175 | legumain (Lgmn) | 0.0000 | 1.20 |
| AK080408 | 7 days neonate cerebellum cDNA, RIKEN full-length enriched library, | 0.0000 | -1.19 |
| AK047739 | adult male corpus striatum cDNA, RIKEN full-length enriched library, | 0.0000 | -1.19 |
| AK033502 | adult male colon cDNA, RIKEN full-length enriched library, | 0.0000 | -1.19 |
| AK086604 | 15 days embryo head cDNA, RIKEN full-length enriched library, | 0.0000 | -1.19 |
| NM_007643 | CD36 antigen (Cd36) | 0.0000 | -1.18 |
| NM_031167 | interleukin 1 receptor antagonist (Il1rn), transcript variant 1 | 0.0000 | 1.18 |
| NM_178929 | Kazal-type serine peptidase inhibitor domain 1 (Kazald1) | 0.0000 | -1.17 |
| NM_026055 | ribosomal protein L39 (Rpl39) | 0.0000 | -1.17 |
| BC062904 | UBX domain containing 7, mRNA (cDNA clone IMAGE:6812948) | 0.0000 | -1.16 |
| AA733629 | AA733629 vu74b08.r1 Stratagene mouse skin (#937313) | 0.0000 | 1.16 |
| AK049588 | 7 days embryo whole body cDNA, RIKEN full-length enriched library, | 0.0000 | -1.16 |
| NM_028055 | RIKEN cDNA 1500005I02 gene (1500005I02Rik) | 0.0000 | 1.15 |
| NM_028276 | UTP14, U3 small nucleolar ribonucleoprotein, homolog A (yeast) (Utp14a) | 0.0000 | -1.15 |
| TC1496338 | Unknown | 0.0000 | -1.15 |
| NM_011635 | tumor rejection antigen P1A (Trap1a) | 0.0000 | -1.14 |
| NM_172267 | phytanoyl-CoA dioxygenase domain containing 1 (Phyhd1) | 0.0000 | 1.14 |
| NAP031415-1 | Unknown | 0.0000 | -1.14 |
| NM_008682 | neural precursor cell expressed, developmentally down-regulated gene 1 (Nedd1) | 0.0000 | -1.14 |
| NM_001003934 | reticulon 3 (Rtn3), transcript variant 1 | 0.0000 | -1.12 |
| NAP045842-1 | Unknown | 0.0000 | 1.11 |
| NM_199476 | ribonucleotide reductase M2 B (TP53 inducible) (Rrm2b) | 0.0000 | -1.11 |
| AK040293 | 0 day neonate thymus cDNA, RIKEN full-length enriched library, | 0.0000 | -1.09 |
| NM_001033157 | RIKEN cDNA 5730507C01 gene (5730507C01Rik) | 0.0000 | -1.08 |
| NM_010163 | exostoses (multiple) 2 (Ext2) | 0.0000 | -1.08 |
| AK011044 | 13 days embryo liver cDNA, RIKEN full-length enriched library, | 0.0000 | -1.07 |
| AK042944 | 7 days neonate cerebellum cDNA, RIKEN full-length enriched library, | 0.0000 | 1.07 |
| AK122354 | mRNA for mKIAA0717 protein | 0.0000 | 1.06 |
| NM_146875 | olfactory receptor 895 (Olfr895) | 0.0000 | -1.06 |
| NM_133245 | erythroid associated factor (Eraf) | 0.0069 | 1.62 |
| AK087319 | 0 day neonate lung cDNA, RIKEN full-length enriched library, | 0.0069 | 1.42 |
| NM_031254 | triggering receptor expressed on myeloid cells 2 (Trem2) | 0.0069 | 1.40 |
| NM_053113 | eosinophil-associated, ribonuclease A family, member 11 (Ear11) | 0.0069 | -1.39 |
| NM_022435 | trans-acting transcription factor 5 (Sp5) | 0.0069 | 1.38 |
| NM_007529 | brevican (Bcan) | 0.0069 | -1.38 |
| AK054424 | 2 days pregnant adult female ovary cDNA, RIKEN full-length enriched library, | 0.0069 | -1.32 |
| NM_175362 | caspase recruitment domain family, member 11 (Card11) | 0.0069 | -1.32 |
| NM_009397 | tumor necrosis factor, alpha-induced protein 3 (Tnfaip3) | 0.0069 | 1.30 |
| NM_172833 | mucosa associated lymphoid tissue lymphoma translocation gene 1 (Malt1) | 0.0069 | 1.29 |
| NM_008690 | nuclear factor of kappa light polypeptide gene enhancer in B-cells inhibitor, epsilon (Nfkbie) | 0.0069 | 1.29 |
| NM_133775 | RIKEN cDNA 9230117N10 gene (9230117N10Rik) | 0.0069 | 1.28 |
| NM_009853 | CD68 antigen (Cd68) | 0.0069 | 1.28 |
| NM_025326 | RIKEN cDNA 0610011I04 gene (0610011I04Rik) | 0.0069 | 1.27 |
| NM_172690 | expressed sequence N28178 (N28178) | 0.0069 | -1.25 |
| NM_007464 | baculoviral IAP repeat-containing 3 (Birc3) | 0.0069 | 1.25 |
| NM_011819 | growth differentiation factor 15 (Gdf15) | 0.0069 | 1.24 |
| NM_009982 | cathepsin C (Ctsc) | 0.0069 | 1.21 |
| AF459018 | complement component C1RB (C1rb) mRNA, complete cds. | 0.0069 | 1.20 |
| NM_008352 | interleukin 12b (Il12b), _008352] | 0.0069 | 1.20 |
| NM_030707 | macrophage scavenger receptor 2 (Msr2) | 0.0069 | 1.20 |
| NM_001001326 | suppression of tumorigenicity 5 (St5), transcript variant 1 | 0.0069 | 1.18 |
| NM_024277 | ribosomal protein S27a (Rps27a), transcript variant 1 | 0.0069 | -1.18 |
| AK079958 | adult male aorta and vein cDNA, RIKEN full-length enriched library, | 0.0069 | -1.16 |
| NM_009434 | pleckstrin homology-like domain, family A, member 2 (Phlda2) | 0.0069 | -1.14 |
| NM_007656 | CD82 antigen (Cd82) | 0.0069 | 1.14 |
| AK014771 | 0 day neonate head cDNA, RIKEN full-length enriched library | 0.0069 | -1.14 |
| AK020576 | adult male urinary bladder cDNA, RIKEN full-length enriched library | 0.0069 | 1.13 |
| NM_019986 | hyaluronic acid binding protein 4 (Habp4) | 0.0069 | -1.11 |
| NM_178719 | Smith-Magenis syndrome chromosome region, candidate 7-like (human) (Smcr7l) | 0.0069 | -1.10 |
| NM_199303 | bactericidal/permeability-increasing protein-like 3 (Bpil3) | 0.0069 | 1.08 |
| NM_146349 | olfactory receptor 1128 (Olfr1128) | 0.0069 | 1.08 |
| NM_177471 | coiled-coil domain containing 69 (Ccdc69) | 0.0069 | 1.08 |
| AK042586 | 7 days neonate cerebellum cDNA, RIKEN full-length enriched library | 0.0069 | -1.06 |
| BC089618 | cDNA clone MGC:107680 IMAGE:6766535 | 0.0124 | 1.59 |
| NM_144938 | complement component 1, s subcomponent (C1s) | 0.0124 | 1.34 |
| NM_021704 | chemokine (C-X-C motif) ligand 12 (Cxcl12), transcript variant 1 | 0.0124 | 1.34 |
| NM_016900 | caveolin 2 (Cav2) | 0.0124 | -1.26 |
| NM_019696 | carboxypeptidase X 1 (M14 family) (Cpxm1) | 0.0124 | 1.26 |
| NM_145942 | 3-hydroxy-3-methylglutaryl-Coenzyme A synthase 1 (Hmgcs1) | 0.0124 | 1.24 |
| NM_007584 | discoidin domain receptor family, member 1 (Ddr1) | 0.0124 | 1.23 |
| NM_172641 | RIKEN cDNA 9930023K05 gene (9930023K05Rik) | 0.0124 | 1.21 |
| NM_207214 | exocyst complex component 5 (Exoc5) | 0.0124 | -1.18 |
| AK040601 | 0 day neonate thymus cDNA, RIKEN full-length enriched library | 0.0124 | -1.17 |
| NM_025834 | protein Z, vitamin K-dependent plasma glycoprotein (Proz) | 0.0124 | 1.16 |
| NM_009779 | complement component 3a receptor 1 (C3ar1) | 0.0124 | 1.15 |
| NM_028176 | cytidine deaminase (Cda) | 0.0124 | -1.15 |
| NM_027007 | zinc finger protein 397 (Zfp397) | 0.0124 | -1.14 |
| XM_132808 | PREDICTED: RIKEN cDNA 2510049J12 gene, transcript variant 1 (2510049J12Rik) | 0.0124 | 1.12 |
| BC043115 | RIKEN cDNA 0610010D24 gene, mRNA (cDNA clone MGC:58041 IMAGE:6408630) | 0.0124 | 1.12 |
| NM_011289 | ribosomal protein L27 (Rpl27) | 0.0124 | -1.11 |
| NM_052993 | core 1 UDP-galactose:N-acetylgalactosamine-alpha-R beta 1,3-galactosyltransferase (C1galt1) | 0.0124 | -1.09 |
| AK054250 | 2 days pregnant adult female ovary cDNA, RIKEN full-length enriched library | 0.0124 | 1.07 |
| NM_177280 | RIKEN cDNA B230206H07 gene (B230206H07Rik) | 0.0124 | -1.07 |
| NM_146828 | olfactory receptor 975 (Olfr975) | 0.0124 | -1.06 |
| NM_020597 | beta-microseminoprotein (Msmb) | 0.0124 | -1.05 |
| NM_029362 | chromatin modifying protein 4B (Chmp4b) | 0.0164 | 1.73 |
| NM_021443 | chemokine (C-C motif) ligand 8 (Ccl8) | 0.0164 | 1.70 |
| NM_020509 | resistin like alpha (Retnla) | 0.0164 | 1.54 |
| NM_020001 | C-type lectin domain family 4, member n (Clec4n) | 0.0164 | 1.40 |
| NM_013521 | formyl peptide receptor 1 (Fpr1) | 0.0164 | -1.34 |
| NM_013589 | latent transforming growth factor beta binding protein 2 (Ltbp2) | 0.0164 | 1.33 |
| NM_054098 | STEAP family member 4 (Steap4) | 0.0164 | 1.31 |
| NM_010207 | fibroblast growth factor receptor 2 (Fgfr2), transcript variant 1 | 0.0164 | 1.29 |
| AK037357 | 16 days neonate thymus cDNA, RIKEN full-length enriched library | 0.0164 | 1.25 |
| NM_009052 | brain expressed gene 1 (Bex1) | 0.0164 | -1.24 |
| AK020062 | 13 days embryo male testis cDNA, RIKEN full-length enriched library | 0.0164 | -1.24 |
| NM_013598 | kit ligand (Kitl) | 0.0164 | -1.23 |
| NM_138656 | mevalonate (diphospho) decarboxylase (Mvd) | 0.0164 | 1.21 |
| NM_025326 | RIKEN cDNA 0610011I04 gene (0610011I04Rik) | 0.0164 | 1.19 |
| AK164516 | 13 days embryo heart cDNA, RIKEN full-length enriched library | 0.0164 | -1.16 |
| NM_009169 | split hand/foot malformation (ectrodactyly) type 1 (Shfm1) | 0.0164 | -1.15 |
| NM_024229 | phosphate cytidylyltransferase 2, ethanolamine (Pcyt2) | 0.0164 | 1.15 |
| NM_144515 | zinc finger protein 52 (Zfp52) | 0.0164 | -1.15 |
| NM_011174 | proline rich protein HaeIII subfamily 1 (Prh1) | 0.0164 | -1.14 |
| AK050947 | 9 days embryo whole body cDNA, RIKEN full-length enriched library | 0.0164 | 1.08 |
| AK031780 | 11 days embryo head cDNA, RIKEN full-length enriched library | 0.0164 | -1.07 |
| AV231787 | AV231787 RIKEN full-length enriched, 0 day neonate skin cDNA clone 4632409J21 3 | 0.0164 | 1.07 |
| XM_894811 | PREDICTED: keratin associated protein 3-1 (Krtap3-1) | 0.0164 | 1.07 |
| AK048083 | 16 days embryo head cDNA, RIKEN full-length enriched library | 0.0164 | -1.06 |
| NM_182714 | olfactory receptor 91 (Olfr91) | 0.0164 | 1.05 |
| AK030827 | adult male thymus cDNA, RIKEN full-length enriched library | 0.0210 | 1.45 |
| NM_009789 | S100 calcium binding protein G (S100g) | 0.0210 | -1.38 |
| NM_011313 | S100 calcium binding protein A6 (calcyclin) (S100a6) | 0.0210 | -1.22 |
| AK049998 | adult male hippocampus cDNA, RIKEN full-length enriched library | 0.0210 | 1.20 |
| NM_031195 | macrophage scavenger receptor 1 (Msr1) | 0.0210 | 1.19 |
| NM_010188 | Fc receptor, IgG, low affinity III (Fcgr3) | 0.0210 | 1.17 |
| AK012052 | 10 days embryo whole body cDNA, RIKEN full-length enriched library | 0.0210 | -1.16 |
| AK083469 | 9 days embryo whole body cDNA, RIKEN full-length enriched library | 0.0210 | -1.13 |
| NM_001003910 | Grp94 neighboring nucleotidase variant 4 (Gnn), transcript variant 3 | 0.0210 | 1.11 |
| AK077997 | 14 days embryo thymus cDNA, RIKEN full-length enriched library | 0.0210 | -1.11 |
| NM_020020 | melanoma antigen, family A, 8 (Magea8) | 0.0210 | -1.10 |
| NM_009917 | chemokine (C-C motif) receptor 5 (Ccr5) | 0.0243 | 1.44 |
| AK005562 | adult female placenta cDNA, RIKEN full-length enriched library | 0.0243 | 1.43 |
| AK172137 | activated spleen cDNA, RIKEN full-length enriched library | 0.0243 | 1.41 |
| NM_025359 | tetraspanin 13 (Tspan13) | 0.0243 | -1.30 |
| NM_146189 | myosin binding protein C, fast-type (Mybpc2) | 0.0243 | 1.23 |
| NM_011593 | tissue inhibitor of metalloproteinase 1 (Timp1) | 0.0243 | 1.20 |
| NM_009952 | cAMP responsive element binding protein 1 (Creb1), transcript variant B | 0.0243 | -1.20 |
| AK031720 | 13 days embryo male testis cDNA, RIKEN full-length enriched library | 0.0243 | -1.19 |
| NM_033218 | sterol regulatory element binding factor 2 (Srebf2) | 0.0243 | 1.18 |
| NM_173371 | hexose-6-phosphate dehydrogenase (glucose 1-dehydrogenase) (H6pd) | 0.0243 | 1.18 |
| NM_007778 | colony stimulating factor 1 (macrophage) (Csf1) | 0.0243 | 1.16 |
| BC059053 | RIKEN cDNA 5730445M16 gene, mRNA (cDNA clone IMAGE:6821066) | 0.0243 | -1.15 |
| NM_147201 | nuclear receptor binding protein (Nrbp) | 0.0243 | -1.14 |
| NM_001012309 | coiled-coil domain containing 55 (Ccdc55) | 0.0243 | -1.09 |
| AK016216 | adult male testis cDNA, RIKEN full-length enriched library | 0.0243 | -1.09 |
| NM_016714 | nucleoporin 50 (Nup50) | 0.0243 | 1.09 |
| AK047975 | 16 days embryo head cDNA, RIKEN full-length enriched library | 0.0243 | 1.07 |
| AK033492 | adult male colon cDNA, RIKEN full-length enriched library | 0.0243 | 1.07 |
| BC006942 | cDNA clone MGC:6909 IMAGE:2655941, complete cds | 0.0243 | -1.06 |
| AK077972 | 13 days embryo male testis cDNA, RIKEN full-length enriched library | 0.0243 | 1.06 |
| NM_009046 | avian reticuloendotheliosis viral (v-rel) oncogene related B (Relb) | 0.0280 | 1.34 |
| NM_007535 | B-cell leukemia/lymphoma 2 related protein A1c (Bcl2a1c) | 0.0280 | 1.22 |
| NM_172769 | sterol-C5-desaturase (fungal ERG3, delta-5-desaturase) homolog (S. cerevisae) (Sc5d) | 0.0280 | 1.18 |
| NM_009399 | tumor necrosis factor receptor superfamily, member 11a (Tnfrsf11a) | 0.0280 | 1.18 |
| XM_484069 | PREDICTED: similar to 60S ribosomal protein L17 (L23) (Amino acid starvation-induced protein) | 0.0280 | -1.17 |
| BC062654 | GNAS (guanine nucleotide binding protein, alpha stimulating) complex locus | 0.0280 | -1.14 |
| NM_023517 | tumor necrosis factor (ligand) superfamily, member 13 (Tnfsf13) | 0.0280 | 1.13 |
| XM_975146 | PREDICTED: RIKEN cDNA B230369F24 gene (B230369F24Rik) | 0.0280 | -1.13 |
| NM_145583 | FGF receptor activating protein 1 (Frag1) | 0.0280 | -1.10 |
| AK048715 | 0 day neonate cerebellum cDNA, RIKEN full-length enriched library | 0.0280 | -1.09 |
| NM_010703 | lymphoid enhancer binding factor 1 (Lef1) | 0.0315 | -1.26 |
| NM_009820 | runt related transcription factor 2 (Runx2) | 0.0315 | -1.25 |
| XM_885873 | PREDICTED: bromodomain adjacent to zinc finger domain 1A (Baz1a) | 0.0315 | -1.24 |
| NM_023056 | RIKEN cDNA 1810009M01 gene (1810009M01Rik) | 0.0315 | 1.23 |
| NM_008965 | prostaglandin E receptor 4 (subtype EP4) (Ptger4) | 0.0315 | 1.19 |
| AK010630 | ES cells cDNA, RIKEN full-length enriched library, clone:2410038B05 product:unclassifiable | 0.0315 | -1.13 |
| NM_033561 | Williams-Beuren syndrome chromosome region 1 homolog (human) (Wbscr1) | 0.0315 | -1.11 |
| NM_010103 | EGF-like repeats and discoidin I-like domains 3 (Edil3), transcript variant 2 | 0.0315 | -1.10 |
| NM_009490 | vomeronasal 2, receptor, 15 (V2r15) | 0.0315 | -1.08 |
| NM_146854 | olfactory receptor 982 (Olfr982) | 0.0315 | -1.07 |
| NM_178656 | RIKEN cDNA A530088H08 gene (A530088H08Rik) | 0.0315 | 1.07 |
| NM_023785 | chemokine (C-X-C motif) ligand 7 (Cxcl7) | 0.0340 | -1.50 |
| NM_013769 | tight junction protein 3 (Tjp3) | 0.0340 | 1.34 |
| NM_030612 | nuclear factor of kappa light polypeptide gene enhancer in B-cells inhibitor, zeta (Nfkbiz) | 0.0340 | 1.28 |
| NM_007860 | deiodinase, iodothyronine, type I (Dio1) | 0.0340 | 1.22 |
| XM_139711 | PREDICTED: AT rich interactive domain 1B (Swi1 like), transcript variant 1 (Arid1b) | 0.0340 | -1.19 |
| NM_010071 | docking protein 2 (Dok2) | 0.0340 | 1.19 |
| BC048564 | RIKEN cDNA 4930415O20 gene, mRNA (cDNA clone MGC:58575 IMAGE:6705104)4] | 0.0340 | 1.18 |
| NM_183186 | checkpoint suppressor 1 (Ches1) | 0.0340 | -1.17 |
| NM_025904 | RIKEN cDNA 1600012F09 gene | 0.0340 | -1.16 |
| AK049933 | adult male hippocampus cDNA, RIKEN full-length enriched library | 0.0340 | -1.16 |
| NM_177185 | RIKEN cDNA D130059P03 gene | 0.0340 | -1.14 |
| NM_001013808 | similar to RIKEN cDNA 6330416L07 gene (LOC433801) | 0.0340 | -1.13 |
| NM_178746 | RIKEN cDNA 9130023D20 gene (9130023D20Rik) | 0.0340 | -1.12 |
| BC011413 | mRNA similar to ribosomal protein S20 (cDNA clone MGC:6876 IMAGE:2651405) | 0.0340 | -1.12 |
| NM_009094 | ribosomal protein S4, X-linked (Rps4x) | 0.0340 | -1.11 |
| NM_178723 | zinc finger protein 533 (Zfp533) | 0.0340 | -1.08 |
| NM_177132 | RIKEN cDNA 1520401A03 gene (1520401A03Rik) | 0.0340 | 1.07 |
| AK007475 | 10 day old male pancreas cDNA, RIKEN full-length enriched library | 0.0340 | 1.07 |
| NM_010191 | farnesyl diphosphate farnesyl transferase 1 (Fdft1) | 0.0368 | 1.30 |
| NM_007542 | biglycan (Bgn) | 0.0368 | 1.26 |
| NM_007806 | cytochrome b-245, alpha polypeptide (Cyba) | 0.0368 | 1.24 |
| NAP028816-1 | Unknown | 0.0368 | 1.18 |
| XM_991662 | PREDICTED: N-acetyltransferase 12, transcript variant 3 (Nat12) | 0.0368 | -1.17 |
| NM_029813 | RIKEN cDNA 2210418O10 gene (2210418O10Rik), transcript variant 1 | 0.0368 | -1.16 |
| NM_007778 | colony stimulating factor 1 (macrophage) (Csf1) | 0.0368 | 1.15 |
| AK173243 | mRNA for mKIAA1725 protein | 0.0368 | -1.14 |
| NM_029985 | leucine rich repeat containing 42 (Lrrc42) | 0.0368 | 1.13 |
| AK032374 | adult male olfactory brain cDNA, RIKEN full-length enriched library | 0.0368 | -1.13 |
| NM_025598 | RIKEN cDNA 2700038C09 gene (2700038C09Rik) | 0.0368 | -1.10 |
| NM_181750 | R3H domain 1 (binds single-stranded nucleic acids) (R3hdm1) | 0.0368 | -1.10 |
| NM_144910 | CCR4-NOT transcription complex, subunit 6-like (Cnot6l), transcript variant 1 | 0.0368 | -1.10 |
| AK041666 | 3 days neonate thymus cDNA, RIKEN full-length enriched library | 0.0368 | -1.06 |
| NM_009139 | chemokine (C-C motif) ligand 6 (Ccl6) | 0.0393 | 1.31 |
| NM_030165 | chondroitin sulfate GalNAcT-2 (Galnact2) | 0.0393 | -1.22 |
| NM_177000 | RIKEN cDNA C130050O18 gene (C130050O18Rik) | 0.0393 | -1.20 |
| NM_011798 | chemokine (C motif) receptor 1 (Xcr1) | 0.0393 | 1.18 |
| NM_134250 | hepatitis A virus cellular receptor 2 (Havcr2) | 0.0393 | 1.17 |
| AK030538 | adult male pituitary gland cDNA, RIKEN full-length enriched library | 0.0393 | -1.16 |
| NM_019388 | CD86 antigen (Cd86) | 0.0393 | 1.12 |
| XR_002113 | PREDICTED: similar to serine (or cysteine) peptidase inhibitor, clade A, member 3B (LOC667984) | 0.0393 | 1.11 |
| NM_145441 | UBX domain containing 4 (Ubxd4) | 0.0393 | -1.10 |
| L23108 | CD36 antigen mRNA, complete cds | 0.0393 | -1.10 |
| NM_177875 | RIKEN cDNA C130026L21 gene (C130026L21Rik) | 0.0393 | -1.09 |
| A_52_P1173798 | Unknown | 0.0393 | 1.08 |
| NM_011852 | 2'-5' oligoadenylate synthetase 1G (Oas1g) | 0.0393 | -1.07 |
| ENSMUST00000077152 | PREDICTED: similar to olfactory receptor 1289 (LOC623583) | 0.0393 | -1.07 |
| NM_008039 | formyl peptide receptor, related sequence 2 (Fpr-rs2) | 0.0413 | -1.37 |
| NM_178053 | N-acetylglutamate synthase (Nags), transcript variant 2 | 0.0413 | 1.23 |
| BC010322 | histocompatibility 2, class II antigen A, beta 1, mRNA (cDNA clone MGC:6297 IMAGE:2651058) | 0.0413 | 1.23 |
| AK122421 | mRNA for mKIAA1028 protein | 0.0413 | 1.19 |
| NM_033622 | tumor necrosis factor (ligand) superfamily | 0.0413 | 1.19 |
| NM_025286 | solute carrier family 31, member 2 (Slc31a2) | 0.0413 | 1.18 |
| NM_029422 | transmembrane 7 superfamily member 4 (Tm7sf4) | 0.0413 | 1.17 |
| NM_008406 | inter-alpha trypsin inhibitor, heavy chain 1 (Itih1) | 0.0413 | 1.15 |
| NM_023627 | myo-inositol 1-phosphate synthase A1 (Isyna1) | 0.0413 | 1.15 |
| NM_008255 | 3-hydroxy-3-methylglutaryl-Coenzyme A reductase (Hmgcr) | 0.0413 | 1.14 |
| NM_009092 | ribosomal protein S17 (Rps17) | 0.0413 | -1.13 |
| NM_016738 | ribosomal protein L13 (Rpl13) | 0.0413 | -1.12 |
| NM_029364 | glucosamine (N-acetyl)-6-sulfatase (Gns) | 0.0413 | 1.11 |
| NM_175493 | G protein-coupled receptor 68 (Gpr68) | 0.0413 | 1.11 |
| AK035515 | adult male urinary bladder cDNA, RIKEN full-length enriched library | 0.0413 | 1.11 |
| NM_028412 | CDKN1A interacting zinc finger protein 1 (Ciz1) | 0.0413 | -1.10 |
| AK088106 | 2 days neonate thymus thymic cells cDNA, RIKEN full-length enriched library | 0.0413 | 1.06 |
| NM_144943 | CD 207 antigen (Cd207) | 0.0432 | 1.38 |
| NM_011179 | prosaposin (Psap) | 0.0432 | 1.37 |
| NM_009777 | complement component 1, q subcomponent, beta polypeptide (C1qb) | 0.0432 | 1.30 |
| NM_177356 | lysosomal-associated membrane protein 3 (Lamp3) | 0.0432 | 1.24 |
| NM_026515 | RIKEN cDNA 2810417H13 gene (2810417H13Rik) | 0.0432 | 1.23 |
| BC098509 | RIKEN cDNA 3110003A17 gene, mRNA (cDNA clone IMAGE:6432119) | 0.0432 | -1.21 |
| NM_011410 | schlafen 4 (Slfn4) | 0.0432 | -1.21 |
| NM_009083 | ribosomal protein L30 (Rpl30) | 0.0432 | -1.17 |
| NM_172263 | phosphodiesterase 8B (Pde8b) | 0.0432 | -1.16 |
| NM_031185 | A kinase (PRKA) anchor protein (gravin) 12 (Akap12) | 0.0432 | -1.16 |
| NM_009763 | bone marrow stromal cell antigen 1 (Bst1) | 0.0432 | 1.15 |
| NM_172295 | cDNA sequence BC037703 (BC037703) | 0.0432 | 1.14 |
| XM_985954 | PREDICTED: ring finger and KH domain containing 2, transcript variant 1 (Rkhd2) | 0.0432 | -1.13 |
| NM_008969 | prostaglandin-endoperoxide synthase 1 (Ptgs1) | 0.0432 | 1.13 |
| NM_019388 | CD86 antigen (Cd86) | 0.0432 | 1.13 |
| NM_009847 | CD2-associated protein (Cd2ap) | 0.0432 | -1.11 |
| NM_009978 | cystatin 8 (cystatin-related epididymal spermatogenic) (Cst8) | 0.0462 | -1.39 |
| NM_008352 | interleukin 12b (Il12b) | 0.0462 | 1.13 |
| NM_009084 | ribosomal protein L37a (Rpl37a) | 0.0462 | -1.13 |
| XM_898933 | PREDICTED: RIKEN cDNA 4930522O17 gene (4930522O17Rik) | 0.0462 | 1.10 |
| AK007129 | adult male testis cDNA, RIKEN full-length enriched library | 0.0462 | 1.07 |
| AK129036 | mRNA for mKIAA0018 protein | 0.0484 | 1.30 |
| NM_017388 | eosinophil-associated, ribonuclease A family, member 3 (Ear3) | 0.0484 | -1.27 |
| NM_134012 | mbt domain containing 1 (Mbtd1) | 0.0484 | -1.23 |
| NM_013693 | tumor necrosis factor (Tnf) | 0.0484 | 1.23 |
| AK037357 | 16 days neonate thymus cDNA, RIKEN full-length enriched library | 0.0484 | 1.22 |
| NM_145943 | cDNA sequence BC031781 (BC031781) | 0.0484 | -1.18 |
| NM_153159 | zinc finger CCCH type containing 12A (Zc3h12a) | 0.0484 | 1.16 |
| NM_023118 | disabled homolog 2 (Drosophila) (Dab2), transcript variant 1 | 0.0484 | 1.16 |
| NM_130885 | oxidation resistance 1 (Oxr1) | 0.0484 | -1.12 |
| NM_009020 | recombination activating gene 2 (Rag2) | 0.0484 | -1.07 |

*FC: Fold change, values are over matched control

| **Table S2 Results of PCR array** | |  |
| --- | --- | --- |
|  |  |  |
| **Gene Symbol** | **FDR p-value** | **Fold Change** |
| Cxcl1 | 0.00 | 7.11 |
| Ccl2 | 0.00 | 4.20 |
| Xcr1 | 0.00 | 1.74 |
| Cxcl5 | 0.01 | 29.64 |
| Spp1 | 0.01 | 2.02 |
| Ccl6 | 0.02 | 1.85 |
| C3 | 0.02 | 1.72 |
| Ccl22 | 0.02 | 3.44 |
| Ccr4 | 0.02 | 2.17 |
| Ccl3 | 0.02 | 2.24 |
| Ccl12 | 0.03 | 2.00 |
| Ccl9 | 0.03 | 1.72 |
| Tnf | 0.04 | 1.83 |
| Ccl7 | 0.05 | 3.78 |
| Ccl17 | 0.06 | 2.73 |
| Tnfrsf1a | 0.06 | 1.15 |
| Cxcl9 | 0.10 | 2.11 |
| Ccl8 | 0.10 | 1.88 |
| Ccr5 | 0.10 | 1.52 |
| Bcl6 | 0.11 | 1.19 |
| Abcf1 | 0.13 | 1.21 |
| Ccl20 | 0.16 | 2.72 |
| Ccr7 | 0.16 | 1.32 |
| Cxcl12 | 0.16 | 1.19 |
| Il16 | 0.16 | 1.16 |
| Cxcr3 | 0.17 | 1.15 |
| Cxcl10 | 0.18 | 1.57 |
| Cxcl11 | 0.18 | 2.22 |
| Ccl19 | 0.22 | 1.52 |
| Mif | 0.22 | 1.14 |
| Il1r2 | 0.22 | 1.58 |
| Il18 | 0.23 | -1.20 |
| Crp | 0.24 | 1.74 |
| Ccl11 | 0.24 | 1.65 |
| Gusb | 0.24 | 1.26 |
| Tnfrsf1b | 0.24 | 1.11 |
| Itgb2 | 0.24 | 1.09 |
| PPC | 0.24 | 1.09 |
| Il10rb | 0.25 | 1.13 |
| Cd40lg | 0.28 | 1.54 |
| Ccl4 | 0.28 | 1.22 |
| Gapdh | 0.28 | 1.20 |
| Ccr6 | 0.32 | 1.27 |
| Ccr9 | 0.32 | 1.21 |
| Ccr3 | 0.33 | 1.30 |
| Ltb | 0.33 | 1.14 |
| Hsp90ab1 | 0.33 | 1.07 |
| Ifng | 0.34 | -1.16 |
| Tollip | 0.39 | 1.09 |
| Ccl5 | 0.41 | -1.10 |
| Il13ra1 | 0.42 | 1.22 |
| Casp1 | 0.44 | 1.06 |
| Lta | 0.45 | 1.29 |
| Il6st | 0.47 | 1.10 |
| Il10 | 0.51 | 1.29 |
| Il15 | 0.54 | 1.06 |
| Il2rb | 0.59 | 1.08 |
| Il8rb | 0.62 | -1.26 |
| Ccr8 | 0.63 | 1.24 |
| Ccr10 | 0.64 | 1.24 |
| Itgam | 0.65 | 1.12 |
| Il1a | 0.65 | 1.10 |
| Ccr2 | 0.65 | 1.09 |
| PPC | 0.65 | 1.06 |
| RTC | 0.65 | 1.04 |
| Actb | 0.66 | -1.03 |
| Pf4 | 0.68 | 1.10 |
| Il1r1 | 0.68 | 1.04 |
| Il10ra | 0.68 | -1.04 |
| Hprt1 | 0.68 | 1.03 |
| Cxcl13 | 0.69 | 1.49 |
| Il1b | 0.72 | -1.19 |
| Il17b | 0.72 | 1.19 |
| Il11 | 0.72 | 1.13 |
| Il1f8 | 0.72 | 1.12 |
| Ccr1 | 0.72 | 1.06 |
| Cx3cl1 | 0.72 | 1.05 |
| Cxcr5 | 0.77 | 1.09 |
| Scye1 | 0.77 | -1.03 |
| RTC | 0.77 | 1.03 |
| Il4 | 0.78 | 1.08 |
| Il1f6 | 0.80 | 1.08 |
| Il13 | 0.80 | 1.07 |
| Il20 | 0.80 | -1.07 |
| Il3 | 0.80 | -1.07 |
| Tgfb1 | 0.80 | 1.02 |
| Cxcl15 | 0.80 | -1.05 |
| MGDC | 0.80 | -1.07 |
| RTC | 0.83 | 1.02 |
| Ccl25 | 0.87 | 1.03 |
| Ccl1 | 0.95 | 1.02 |
| Il5ra | 0.97 | -1.02 |
| Ccl24 | 0.97 | 1.01 |
| Il6ra | 0.97 | 1.00 |
| PPC | 0.97 | -1.00 |
| Il2rg | 0.97 | -1.00 |
